# Ecosystem restoration in fire-managed savanna woodlands: Effects on biodiversity, local livelihoods and fire intensity

**DOI:** 10.1007/s13280-020-01343-7

**Published:** 2020-05-25

**Authors:** Maria Ulrika Johansson, Firew Bekele Abebe, Sileshi Nemomissa, Tamrat Bekele, Kristoffer Hylander

**Affiliations:** 1 Department of Ecology, Environment and Plant Sciences, Stockholm University, 106 91 Stockholm, Sweden; 2 Bolin Centre for Climate Research, Stockholm University, 106 91 Stockholm, Sweden; 3 Department of Natural Resources Management, Haramaya University, Dire Dawa, Ethiopia; 4 Department of Plant Biology and Biodiversity Management, Addis Ababa University, PO Box 3434, Addis Ababa, Ethiopia

**Keywords:** Cool fires, Experimental fire, REDD+, Seedling establishment, Shrub encroachment, Traditional fire knowledge

## Abstract

Ethiopia aims to restore 15 million ha degraded forests and woodlands, but effects on the potentially contrasting goals of long-term carbon storage, biodiversity and sustainable livelihoods are unknown. To quantify the effects of grazing exclusion on vegetation and fire behaviour, we established six 30 × 30 m fenced exclosures with grazed controls, in a mesic wooded savanna. Experimental burns were done after 1.5 years. Tree seedlings were few but more common inside fences. Field layer cover and biomass increased inside fences, and grass species increased in numbers and cover. Fire intensity was higher inside fences, killing shrubs and saplings but not mature trees. Interviews confirmed that overgrazing has resulted in “*cool fires*”, causing shrub encroachment. High-intensity fires occurred in the 1980s after a zoonotic disease killed most livestock. Short-term increase in carbon storage through fire and grazing exclusion may lead to loss of pasture, and in the long-term increased wildfire risk.

**Electronic supplementary material:**

The online version of this article (10.1007/s13280-020-01343-7) contains supplementary material, which is available to authorized users.

## Introduction

The UN programme for reducing emissions from deforestation and forest degradation (REDD+) aims to increase carbon capture to mitigate climate change by reducing deforestation in developing countries. Ethiopia pledges to restore 15 million ha of degraded forests and woodlands to sequester carbon, and at the same time protect biodiversity and foster sustainable livelihoods (Lemenih and Kassa 2014). These objectives are not always synergistic, and the role of fire is often misunderstood. The maps used to identify land for forest restoration are based on climatic conditions for tree growth, excluding the fact that fire (natural and anthropogenic) expanded grassy biomes at the expense of forests for millennia (Parr et al. 2014). Savanna woodlands are characterized by a grass-dominated field layer, a mosaic of tree stands with canopy covers ranging 5–80%, and regular surface fires. REDD+, on the other hand, defines tree covers > 10–40% as forest/woodland and often applies a general fire-exclusion policy (Parr et al. 2014). A sudden increase in tree canopy in savanna woodlands may threaten grassland species, groundwater recharge, and local livelihoods (Veldman et al. 2019). It can also cause fuel build-up and increased fire intensities, top-killing mature trees and jeopardizing long-term carbon storage (Russell-Smith et al. 2015). In Africa, management of protected areas needs to balance the often contrasting goals of long-term carbon storage, biodiversity and sustainable livelihoods (Leach and Scoones 2015). In East Africa, many protected areas were earlier communal grazing lands managed by fire (Neumann 1998). In climates marginal for agriculture, livestock herding is one of the most resilient food production systems, and the system that most closely resembles non-anthropogenic disturbances of fire and grazing (Sayre et al. 2013). In East Africa, livestock herding has been widespread for at least 7000 years (Marshall and Hildebrand 2002), with traditional fire management as an integral part.

Grazers control species composition, primary production, soil properties, nutrient cycling, and soil microbial communities (McNaughton et al. 1988). Effects on vegetation may depend on herbivore sizes, densities, migration, and foraging selectivity (McNaughton et al. 1988). Overgrazing has been seen as a major cause of rangeland degradation in Africa (Lamprey 1983). It may reduce pasture productivity (Tefera et al. 2007), decrease species diversity (Angassa 2014) and increase soil compaction (Savadogo et al. 2007). Shrub encroachment degrades grasslands globally, and underlying mechanisms have long been discussed. Multiple interactions between rainfall, soils, fire and herbivores control the competitive outcome between grasses and woody species (Eckhardt et al. 2000; Hagenah et al. 2009; Kirkpatrick and Bridle 2016). Fire suppression and restricted herd mobility has been reported to contribute to shrub encroachment (Dalle et al. 2006; Gil-Romera et al. 2011; Angassa 2014). In the last decades, a new understanding of savanna dynamics has emerged, suggesting that they are non-equilibrium systems, governed by long-term variation in precipitation and complex feedbacks, and therefore cannot be managed by static stocking rates based on ideas of equilibrium carrying capacity (Briske et al. 2003).

Savanna woodlands are fire-adapted systems, and mature trees with thick bark and elevated canopies typically survive fires, but saplings and shrubs with thin bark die (Gashaw et al. 2002; Bond and Zaloumis 2016). However, many savanna trees resprout from roots if above-ground parts are fire-killed, facilitating quick regrowth (Gashaw et al. 2002; Schutz et al. 2011). African hunter-gatherers and pastoralists used fire to increase pasture, reduce tree cover and discourage predators and pests (Archibald et al. 2012). From paleorecords, it is hard to differentiate natural from anthropogenic fire regimes, and this is maybe not even relevant in Africa, where man has mastered fire for millennia (Archibald et al. 2012). Anthropogenic fires are typically more early-season, small-scale and low-intensity than climate and fuel-driven fires (Archibald et al. 2012). Therefore, mature trees often survive in anthropogenic fire regimes (Russell-Smith et al. 2015). Frequent burning and grazing for millennia has created cultural landscapes with species assemblages adapted to their specific fire regimes (Archibald et al. 2012). Due to authorities´ misconception of the role of fire, a strict fire-exclusion policy has been implemented across Africa since colonization, with serious consequences for vegetation dynamics and pastoral livelihoods (Laris and Wardell 2006; Angassa and Oba 2008; Butz 2009; Russell and Ward 2016). To increase tree cover in REDD+ projects in Africa, traditional burning and grazing are typically excluded. However, the local effects on vegetation, surface fuels and wildfire risk are largely unknown.

### Aims and objectives

The overall aim is to understand the interactions between fire and grazing, and their effects on long-term carbon storage, biodiversity and local livelihoods in a fire-managed mesic savanna-woodland mosaic. We established a fire- and grazing exclosure experiment in Gibe Sheleko National Park, Ethiopia, and recorded vegetation over 2 years. We quantified effects of grazing exclusion on field layer composition, surface fuel biomass, species composition and tree seedling abundance. We performed experimental burns and measured flame lengths and post-fire shrub mortality. Experiments were done in cooperation with local landusers and complemented by qualitative interviews concerning fire ecology and historical and current management. Based on the results, we suggest a way forward to find synergies between the often contrasting goals of carbon storage, biodiversity conservation and sustainable livelihoods for local agropastoral communities.

## Materials and methods

### Study area

**Fig. 1:**
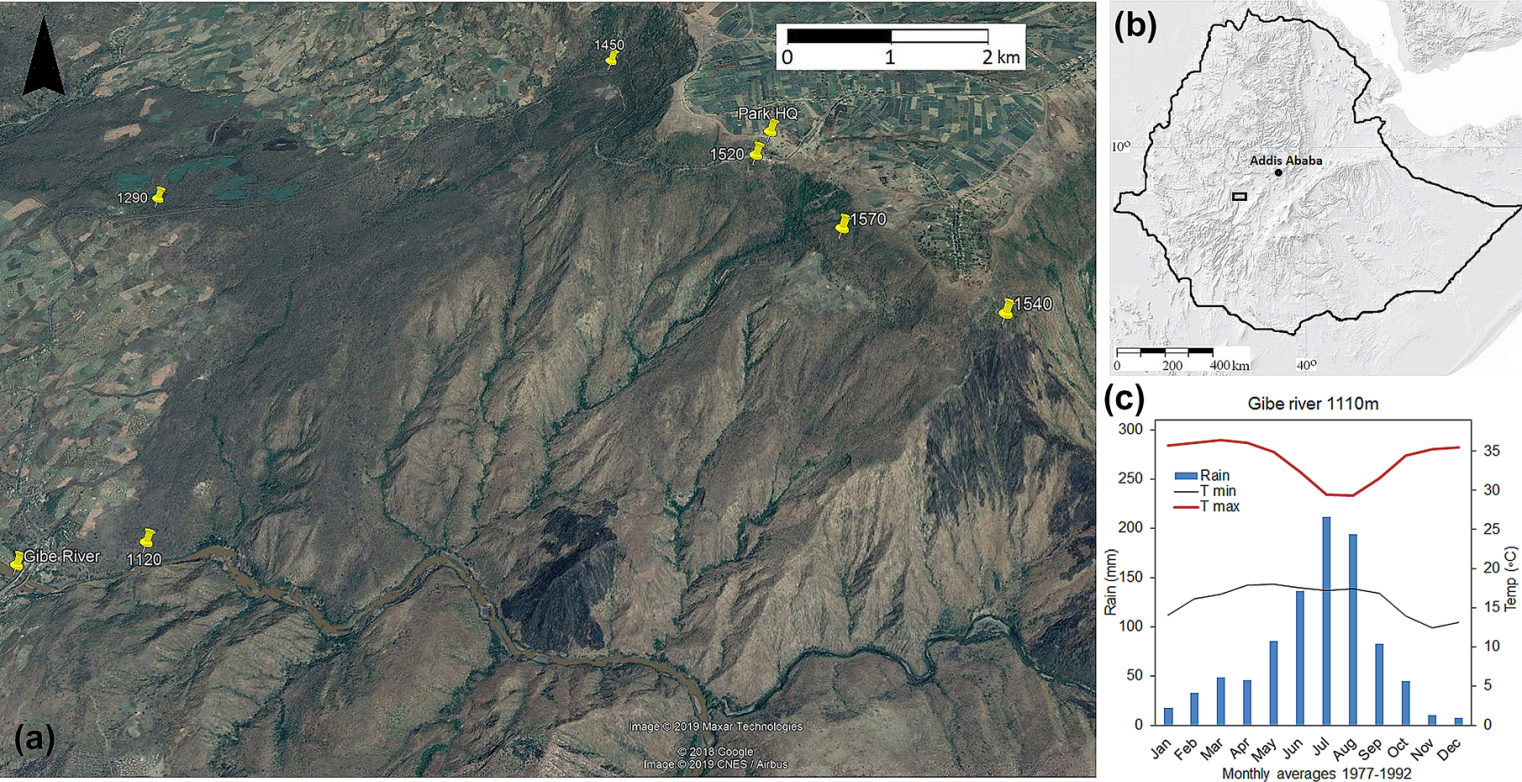
Study area **a** Google Earth™ CNES/Airbus Image of the study area (December 2018) showing the location of exclosures in the landscape, the spatial distribution of higher canopy cover areas and some typical early burn patterns. Exclosure sites labelled with altitude (m a.s.l.). Two large recent burns are visible as black patches just north of Gibe river (left) and Wabe tributary (right). **b** Location in Ethiopia, **c** climate diagram for Gibe River at 1100 m a.s.l., based on ground measurements 1977–1992

The study area is located in a ~ 5 × 10 km area in Gibe Valley National Park (8° 13′ 49″ N, 37° 34′ 38″ E, *Gibe River*, Fig. 1a) in South-Western Ethiopia. The park, established in 2009, is included in the national forest restoration targets, and parts of the park are included in Oromia REDD+. The altitude is ~ 1580 m a.s.l. at the northern plateau (Park HQ, Fig. 1a) and ~ 1100 m a.s.l. at the bridge across Gibe river. Soils are derived from Eocene–Palaeocene basaltic rocks, and soil types are nitisols, acrisols and vertisols (Tadesse et al. 2003). The climate is tropical seasonal, with rainy seasons controlled by two weather systems; the short rains (SE monsoon, March–April) and the long rains (ITCZ in May–October). Average annual rainfall at 1100 m is ~ 920 mm, and average annual max/min temperatures are 34°C and 16°C (Fig. 1c).

**Fig. 2:**
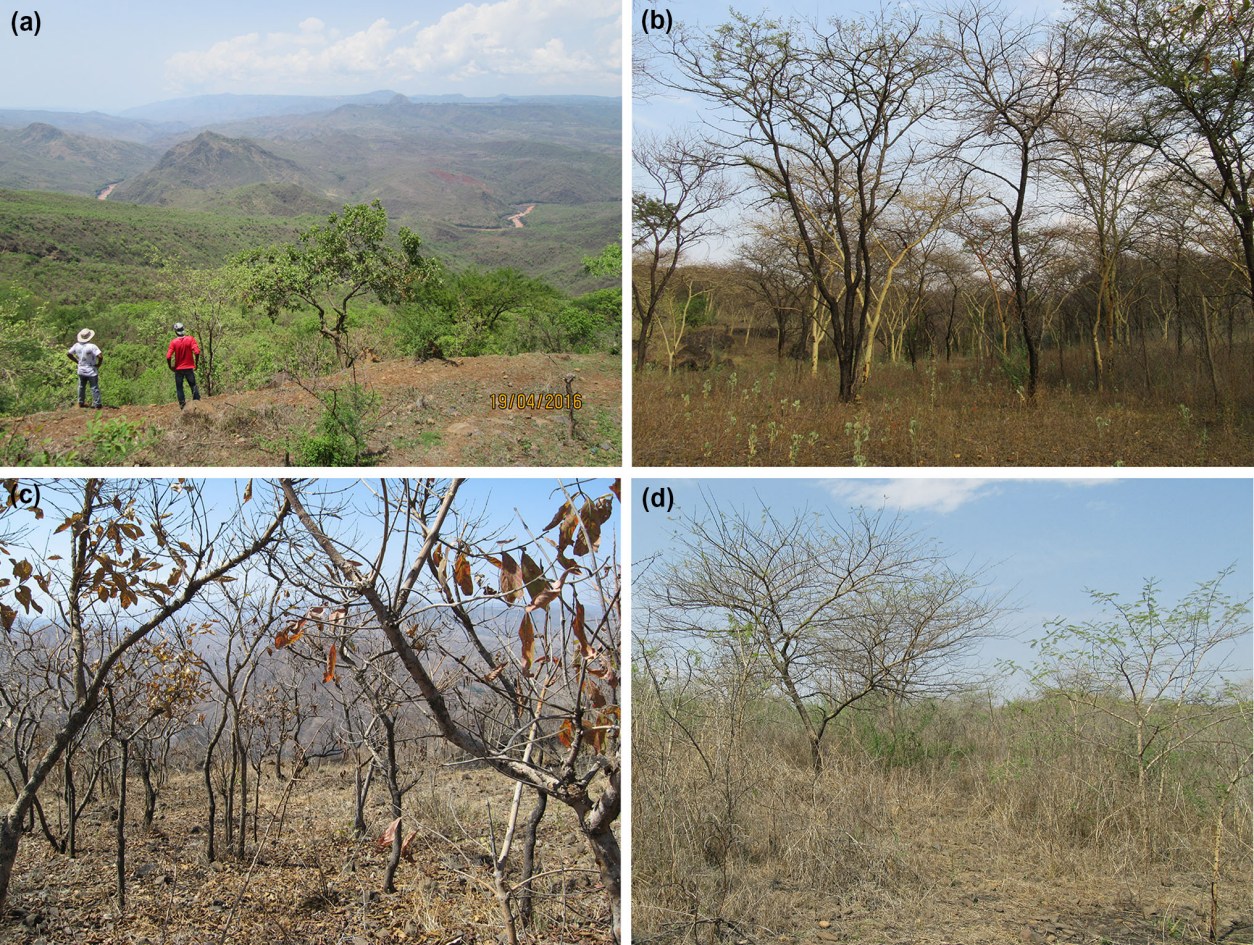
Vegetation characteristics **a** overview of the river gorge from the northern plateau in the rainy season, **b** dry-season acacia woodland, with *Acacia seyal* (green bark) and *A. etebaica* (black bark), **c** combretum woodland with *Combretum collinum* (grey bark) and *C. molle* (black bark), **d** grass-dominated acacia stand with young *Acacia polyacantha* (grey bark) and *A. seyal* (green bark)

The vegetation is dominated by mesic savanna-woodland mosaics, tree canopy covers ranging 0–90%. Narrow strips of evergreen woodlands along streams, and gallery forests along rivers (Fig. 1a). Areas with flat terrain and deeper soils are dominated by deciduous small-leaved nitrogen-fixing trees, like *Acacia polyacantha*, *A. seyal* and *A. etebaica* (Fig. 2b). Stony slopes are dominated by tall savanna grasses (Fig. 2d), or broad-leaved trees like *Combretum molle*, *C. collinum* and *Cussonia holstii* (Fig. 2c). Dominant C4 grass species are *Bothriochloa insculpta*, *Hyparrhenia dregeana* and *H. filipendula*. Common shrubs are *Searsia natalensis* (former *Rhus natalensis*), *Fluegga viros*a and *Dicrostachys cinerea*. Wild herbivores are hippopotamus, greater kudu, Menelik’s bushbuck, bohor reedbuck and olive baboons (Tilahun et al. 2017).

Burning of vegetation is nationally banned, but the river gorge has for long time been under traditional fire management to improve pasture and discourage predators (python, hyena) and to control vector-borne diseases, such as Trypanosomiasis, carried by tse–tse flies. The land is owned by the state, but utilized as communal grazing land. Four different ethnical groups, with different original livelihood strategies, all including cattle, currently utilize the river gorge and a tributary. Human population has fluctuated historically and increased rapidly since the 1990s, partly due to tse–tse control (Reid et al. 1997; Baumgärtner et al. 2008) and resettlement schemes (Reid et al. 1997; Tilahun et al. 2017). Tree cover loss has been substantial for the last 30 years, mainly due to agricultural expansion (Reid et al. 1997; Hailu et al. 2018). People utilize the river gorge for pasture, timber, fuelwood, charcoal and honey production, but tree cutting and grazing have been restricted since park establishment (Tilahun et al. 2017).

### Exclosure establishment

**Fig. 3:**
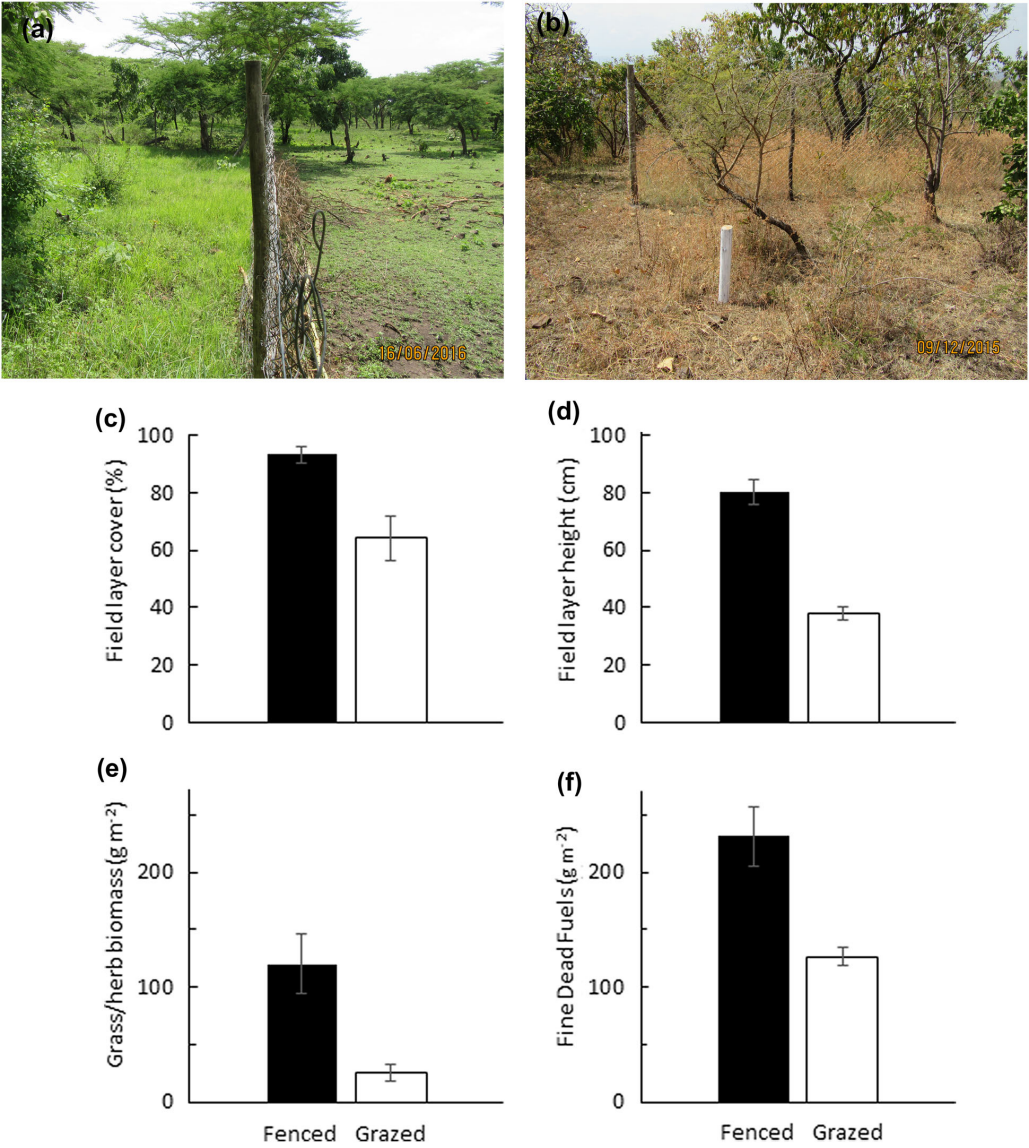
Field layer cover and height in fenced and grazed plots **a** rainy season photo, **b** dry-season photo, **c** average October field layer cover (%), **d** field layer height (cm), **e** average December field layer (grass/herb) biomass (dry weight g m^−2^) f) total fine dead fuel biomass (field layer + leaf litter + fine litter (excl. coarse wood litter), dry weight g m^−2^) in fenced and grazed plots. *n* = 6 sites, Mean ± 1 SE

Permanent exclosures were established in August 2015 at six sites along the altitudinal gradient, from the river (at 1120 m a.s.l.) to just below the plateau (at 1570 m a.s.l.). At each site, a larger woodland stand was selected based on the following criteria: relatively flat ground, without signs of cultivation, no stumps of recently felled trees and with rather homogenous tree canopy. The exclosure was placed randomly and the marked control plot was placed adjacently, 5 m away, with similar slope, soil and tree canopy (Fig. S1). Exclosures were 30 × 30 m, constructed by poles and 180 cm tall strong mesh wire (Fig. 3b), excluding all livestock and large wildlife, except baboons. To exclude unplanned fires, a 20 m wide fire break was burnt annually, using cool backing fires (Fig. S1). A local fence guard was employed at each site to guard and maintain fence and fire breaks until present (2019).

In October 2015, tree canopy cover and shrub canopy cover were quantified in line transects, and baseline data were collected on slope, catena position, soil type, basal area and canopy height (Table S3). Rain data were collected at 1120 and 1600 m a.s.l. (Fig. S2). Monthly soil moisture (Fig. S3) and climate logger data (Fig. S4a, b) were collected at each site.

### Field layer cover and species composition

In 2015, livestock was excluded during half the growing period (which is May–October). In 2016, a full growing season passed before sampling in October–December. To quantify effects of livestock exclusion on field layer cover and species composition, in October 2016 (when many species are in flower/fruit), specimens of all field layer species were collected for identification at the National Herbarium. Area cover of each species was visually estimated in 1 × 1 m quadrats (*n* = 8 per site and treatment, Fig. S1) and field layer height was measured with a measuring stick. Naturally established tree seedlings were also recorded, with notes of species and height.

### Surface fuel biomass

To quantify grazing exclusion effects on dry-season fine dead fuels, we harvested and weighed field layer and litter in December 2016. At 5 sites (since the 1540 m site accidentally burned on December 2nd), we collected all above-ground biomass in 1 × 1 m quadrats (*n* = 5 per site and treatment) and sorted into the following fractions: (1) grass/herb field layer, (2) large leaf litter, (3) wood litter and (4) fine litter, in separate bags. Each fraction was separately weighed (fresh weight, FW) on a field scale (accuracy 0.1 g). Subsamples were transported to the lab for drying in 48°C until constant weight, and dry weight was recorded (DW). Moisture content per dry weight (MC%/DW) was calculated, and this was used to calculate total harvested dry weight in the field (TDW), using the function: TDW = FW/(1 + MC/100). For analyses, we used field layer (grass/herb) biomass separately and total fine dead fuels (pooled field layer + leaf litter + fine litter). Wood litter coarser than 0.63 cm diameter was excluded since it is not part of the fine dead fuels contributing to the flaming front.

### Burning experiments and shrub mortality

We performed burning experiments February 28th–March 3rd, 2017, in the afternoon between 15.00 and 17.00 pm, when wind direction was predominantly upslope. The upslope half of each plot was burnt, giving 4 different treatments per site (Fenced/Burnt, Fenced/Unburnt, Grazed/Burnt and Grazed/Unburnt). We applied fire as upslope head fires, ignited by a drip torch along a 30 m straight ignition line along the lower border (Fig. 5a, b). The control plot at each site was burnt directly after the fenced plot, to have similar weather conditions. Wind speed varied from 0.3 to 1.5 m/s, temperatures from 30 to 35°C and RH from 25 to 43%. Winds were slightly shifting, and short periods of fire line backing against the wind were closely recorded. Maximum flame lengths were determined from photographs (taken every ~ 10 s) at uphill winds of ~ 1–1.5 m/s. For the unobserved fire, flame lengths were estimated from char height on tree trunks. Using the line transects, in August 2017, we recorded shrub above/below-ground post-fire mortality and tree maximum branch kill height, noting species, height and DBH.

### Interview methods

To elucidate landuse history, management objectives and fire knowledge, we did qualitative interviews with 37 residents living inside and close to the park. Eight single-informant, and 10 group interviews, with 2–4 informants, were done October 2015–June 2016. We used semi-structured questionnaires and photos of local vegetation types and different types of fires (Online Appendix S1, ESM S2). Interviews were translated in the field by a local interpreter, and tape-recorded and later transcribed by another interpreter. Responses were entered into a spread sheet with demographic and livelihood data and patterns were sought qualitatively.

### Data analyses

To test the effects of grazing exclusion on field layer cover and height, field layer biomass, and fine fuel biomass, we performed mixed-effect general linear models (GLM) using the R nlme package. Similar models were run for number of species and number of seedlings, using the lme4 package and a Poisson error structure. In all analyses, subplots were nested within treatment and site. Residual plots were inspected to evaluate model assumptions.

Difference in species composition between grazed and fenced plots were tested with an Adonis test using the vegan package, complemented with an indicator species analysis to see which species contributed most to the results (using the labdsv package). To illustrate the variation in species composition among the plots, we ran an NMDS ordination with two axes and default settings in the vegan package.

To test if the proportion of fire-killed shrub shoots/roots differed, we ran a GLM with treatment nested in site as random factor and a binomial error structure. All analyses were made in R 3.3.3 (R Core Team 2017).

## Results

### Grazing exclusion effects on field layer cover and biomass

Livestock exclusion resulted in 45% increase in field layer cover (*p *= 0.020, Fig. 3c) and 115% increase in field layer height (*p *< 0.001, Fig. 3d) in fenced compared to grazed plots (Table S1a). Grass/herb biomass (Fig. 3e) was higher inside exclosures (*p *= 0.025, Table S1b). Total fine fuel biomass was almost twice as high in fenced plots (~ 230 g m^−2^ inside, vs 130 g outside, *p *= 0.047), but with large variation between sites (Fig. 3f, Table S1b).

### Field layer species diversity and natural tree seedling establishment

42 species were identified in the vegetation quadrats (18 herbs, 13 grasses, 7 trees, 4 shrubs and 2 climbers). Numbers of species did not differ between fenced and grazed plots (2.3 vs 3.5/quadrat), but numbers of grass species were higher in fenced plots (1.3 vs 0.6, *p *= 0.008, Table S2). Community composition differed between fenced and grazed plots (*p *= 0.022, Adonis), but differences between sites was larger (Fig. S5). Species contributing most to the difference was the grass *Bothriochloa insculpta*, more abundant in fenced plots, and the herb *Leucas deflexa*, more common in grazed plots (Tables 1, S4).

**Table 1:** Indicator species for fenced and grazed plots, all species with *p *< 0.25 listed). Significant results at the 0.05 level indicated in bold text

Species	Family	Indicator species for	*p* value
*Bothriochloa insculpta*	Poaceae	Fenced	**0.017**
*Heteropogon contortus*	Poaceae	Fenced	0.058
*Sorghum arundinaceum*	Poaceae	Fenced	0.124
*Justicia heterocarpa*	Acanthaceae	Fenced	0.235
*Leucas deflexa*	Lamiaceae	Grazed	**0.041**
*Ageratum conyzoides*	Asteraceae	Grazed	0.074

**Fig. 4:**
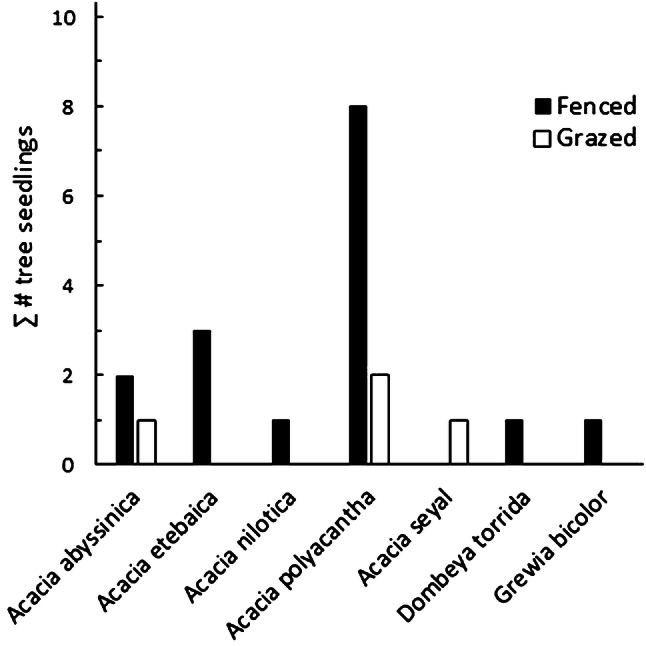
Numbers of naturally established tree seedlings m^−2^ of different species in vegetation quadrats in fenced and grazed plots (October 2016) (*n* = 98 quadrats at 6 sites). Tree seedlings were 5–20 cm tall

We found 16 tree seedlings (6 species) in fenced plots and 4 tree seedlings (3 species) in grazed plots (*p *= 0.013, GLM, Fig. 4).

### Flame lengths, post-fire shrub mortality and branch kill heights

**Fig. 5:**
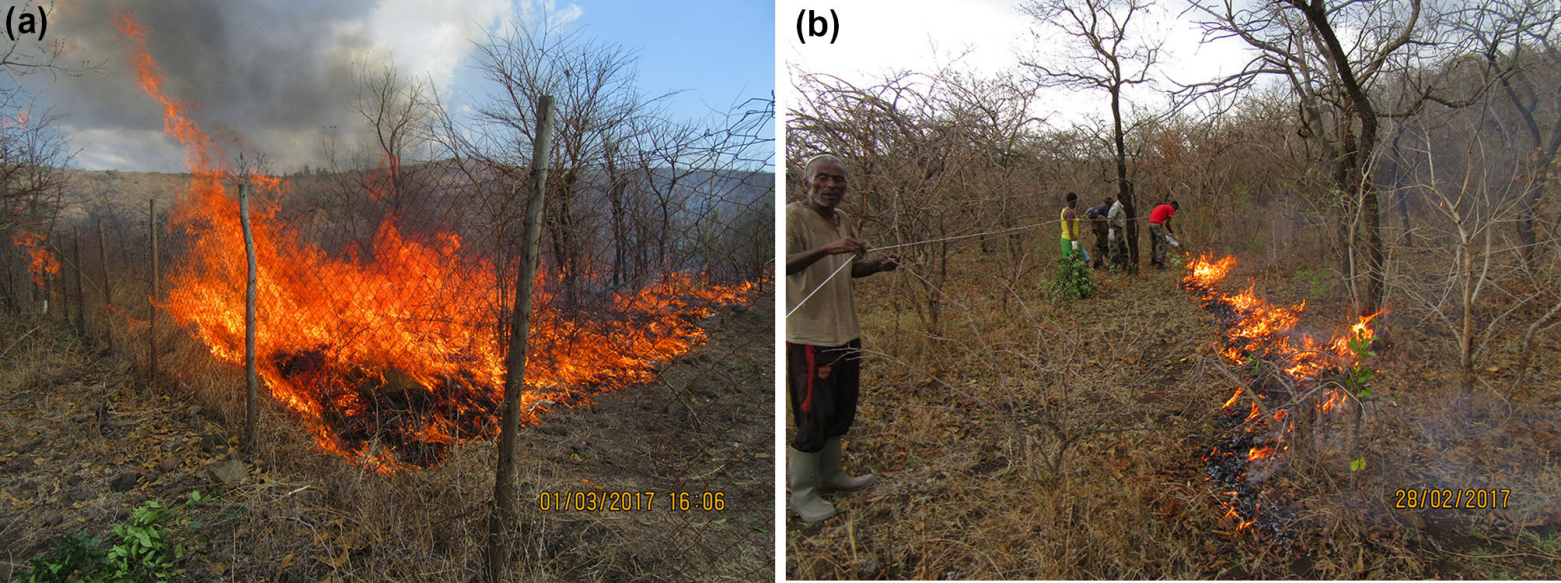
Burning experiments February–March 2017. **a** maximum flame lengths of ca 5.5 m recorded in tall grass fuels inside the fence at the most open-canopy site at 1450 m. **b** Typical flame lengths of 20–40 cm in the broad-leaf litter fuels in the grazed plot

Fire intensity was higher in fenced plots, with maximum flame lengths up to 5.5 m, vs max 0.5 m flames in grazed plots. Average flame lengths at 1 m/s uphill wind speed were 2.2 m in fenced plots vs 0.3 in grazed plots (GLM, *p *= 0.009, Table 2, Fig. 5). There was an exponential relation between surface fuel mass and head-fire flame lengths (at 1 m s^−1^ wind), a doubling in surface fuel biomass (100 → 200 g m^−2^) resulted in ~ 36% increased flame length.

**Table 2:** Burning experiments’ site data, weather data, fire rate of spread (ROS), flame lengths and fireline intensities (FI) in fenced (F) and grazed (G) plots. Burns performed uphill, with the wind. Flame lengths were recorded for head fires at wind speeds ~ 1 m s^−1^. Surface fuels inside fences = mainly cured grass/herb field layer, outside = mainly large *Combretum*-type (broad-leaved) leaf litter

Alt. (masl)	Slope (%, aspect)	Tree canopy type	Treat	Av. fuel load (kg m^−2^)	Av. wind speed (m s^−1^)	Av. T (°C)	Av. RH (%)	Av. ROS (m s^−1^)	Av. flame length (m)	Av. FI^1^ (KJ m^−1^)	Calc. FI^2^ (KJ m^−1^)
1120	4% S	Acacia	F	0.21	1	35	30	0.04	1.2	1790	1259
			G	0.05	1		0		0	No fire	
1290	1% N	Acacia	F	0.20	1	38	24	0.33	0.7	1039	1103
			G	0.14	1	36	24	0.17	0.4	590	386
1450	0.2% S	Acacia	F	0.18	1	37	27	0.67	3.9	5887	2027
			G	0.16	1	32	28	0.27	0.6	889	725
1570	4% W	Broad	F	0.31	1	33	29	0.83	2.6	3909	4335
		Leaved	G	0.11	1	35	29	0.12	0.4	590	223
1520	2% S	Broad	F	0.19	1	33	23	0.67	2.0	2999	2139
		Leaved	G	0.18	1	33	25	0.07	0.2	293	197
1540	3% SE	Broad	F	0.30	1				3.3	Wildfire	
		Leaved	G	0.12	1				0.1	Wildfire	

Moisture content (% DW^−1^) of fine dead fuels = 10–14%. The 1540 m plots burnt in an unplanned fire, and flame lengths were estimated by char heights on tree trunks. The 1120 m grazed plot could not burn due to lack of fuels. FI^1^ = Fireline intensity based on flame lengths using the head-fire equation: FI = 1488.7 L^1.01^ (Clark 1983). FI^2^ calculated using Byram’s (1959) equation: *I* = *H*·*w*·*r*, where *H* = fuel heat content (kJ kg^−1^), *w* = consumed fuel mass (kg m^−2^), and *r* = ROS (m s^−1^). Heat content of grass and litter fuels = 16 890 kJ kg^−1^ (Trollope et al. 1996)

**Fig. 6:**
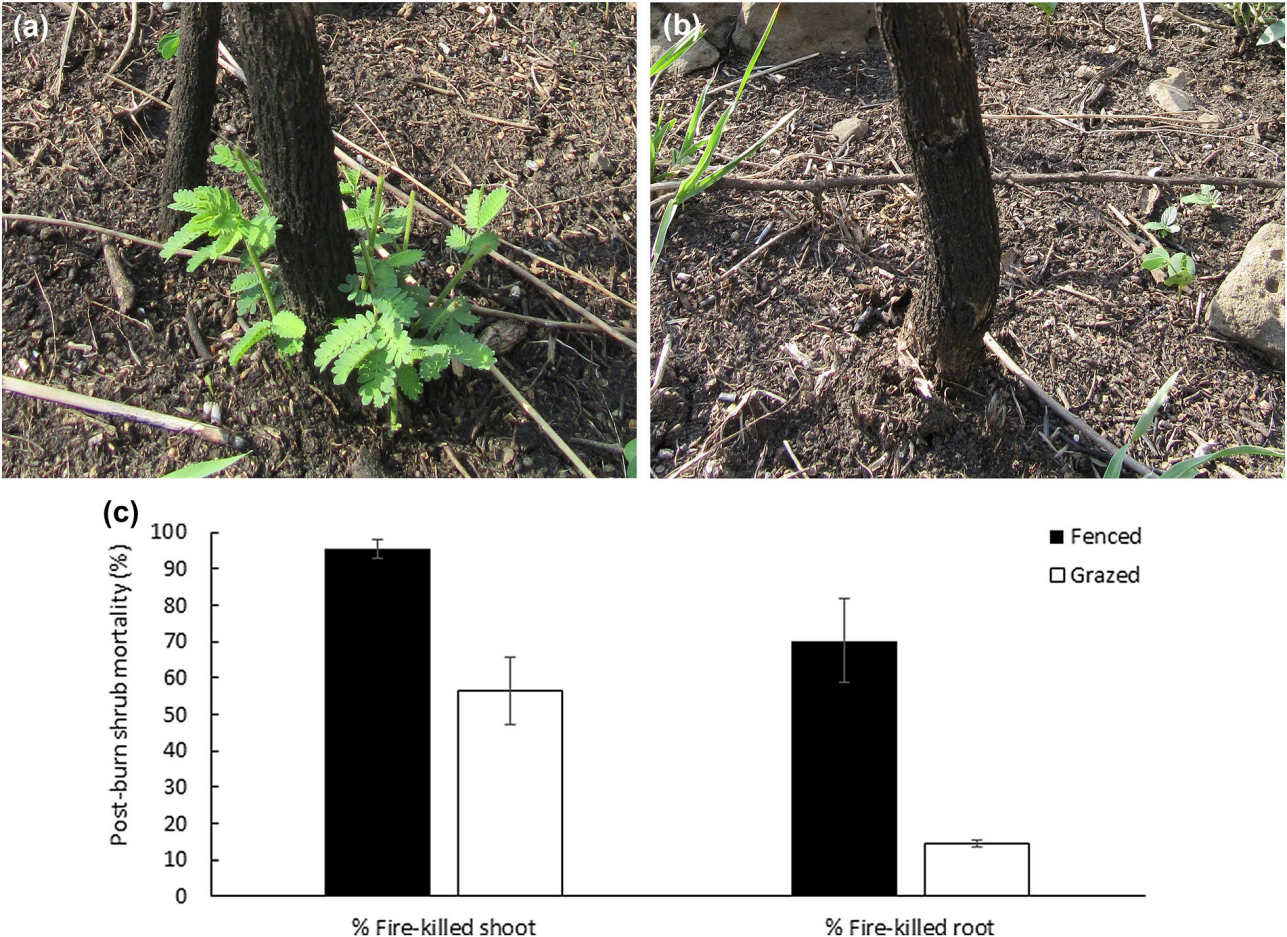
Post-burn shrub mortality **a** average proportion of shrubs with fire-killed shoots, and fire-killed roots, for fenced/grazed burnt treatments. *n* = 6 sites, Mean ± 1 SE. **b** *Dicrostachys cinerea* with fire-killed shoot. **c** *Dicrostachys cinerea* with fire-killed root

Shrubs were mainly ~ 2–3 m tall *Dicrostachys cinerea* (a few *Fluegga virosa* and *Searsia natalensis*). In burnt fenced treatments, 96% of the shrubs were killed above-ground, significantly more than in burnt grazed treatments (57%, GLM, *p *< 0.001, Fig. 6). The proportion of fire-killed roots was higher in burnt fenced, compared to burnt grazed treatments (70% vs 14%, *p *< 0.001, Fig. 6). In unburnt treatments, shrub mortality was zero. *Acacia* saplings (~ 4 years old, 3–4 m height, 3–5 cm DBH, found only in the open-canopy site, Fig. 5a) were top-killed and had a root mortality of 90% in fenced burnt transects, compared to 43% in burnt grazed transects. The highest tree branch kill height was up to 4 m in fenced plots compared to 1 m in grazed plots.

### Smallholder management objectives and vegetation history

Informants were not hesitant to talk about fire management. They said that the river gorge always burned in the dry season. They said that fires are unavoidable and necessary for pasture quality, and for safety by discouraging giant python, lions and hyenas. Informants whose families lived in the area for at least two generations said that in the past their families owned large herds, up to 200 cattle, but there were fewer families then. In the past, residents lived mainly up on the cooler plateau, especially on the north-eastern side. Cattle are important for meat and dairy production, and as draught power for agriculture. Trypanosomiasis disease had not been observed in the area before a first severe epizootic in the early 1980s, which killed almost all livestock. This caused famine and made landusers diversify and intensify agriculture, especially on the north-eastern plateau. Inside the river gorge, the cessation of grazing caused fuel build-up and high-intensity wildfires, which killed even the large *Acacia* trees, according to respondents. After tse–tse eradication programmes in the 1990’s, they started utilizing the river gorge for pasture again. At the same time northern farmers were resettled into the river gorge and investors started small commercial farms. Resettlement ceased with the establishment of the national park in 2009. The landusers want large grass-dominated pastures, and stands of mature flowering trees for honey production, timber and fuelwood. The most unwanted vegetation is spiny scrub which limits livestock access to grass. Elder informants had a deep traditional fire management knowledge, explaining the importance of wind and terrain, and how intensity can be controlled by ignition timing and patterns. All informants said that population growth and the resettlement schemes caused land shortage and overgrazing. Respondents explained that overgrazing reduces the amount of grass, which causes “*cool fires*” (i.e. low-intensity fires with short flames) which are incapable of killing the roots of “*Ader*” (*Dicrostachys cinerea)* which is an invasive shrub. One respondent phrased it like this: “*It is difficult to get rid of Ader by fire, since it easily resprouts from its roots, you need to burn late in April, when it is very dry to be able to kill its roots*”. For more details on interview results see Supporting Information.

## Discussion

We found a strong effect of livestock exclusion on field layer biomass and plant species composition, which has implications for carbon storage, biodiversity and pasture quality. Our findings suggest that high grazing pressure and subsequent cool fires have contributed to shrub encroachment. When grazing was instead excluded for 1.5 years, we show that this resulted in higher fire intensities, killing the shrubs but not trees. Yet, according to our interviews, longer periods of grazing exclusion could cause even more fuel accumulation and high-intensity wildfires killing also mature trees. This would compromise all management goals, leading to the conclusion that some intermediate levels of grazing and fire probably is what should be strived for.

### Field layer cover and species composition

Grazing exclusion increased field layer cover and biomass, and grass species numbers and cover. Some respondents pointed out that the grass inside our exclosures looked like it used to look like earlier, and that this is the preferred pasture status. Long-term studies of community exclosures in Northern Ethiopia show an increase in field layer biomass and species diversity (Mekuria et al. 2018; Gebregergs et al. 2019). We found a small difference in species composition, likely caused by the differential palatability of the species; e.g. the indicator species for grazed plots, *Leucas deflexa* is a common grazing weed in hard-grazed systems (e.g. Kikoti and Mligo 2015). The indicator species for fenced plots, *Bothriochloa insculpta*, is a highly palatable forage grass. In savanna grazing lawns, heavy grazing may increase plant diversity by reducing competition, allowing many weak competitors to thrive (Donaldson et al. 2018). But, constant intense livestock grazing and loss of seasonal migration typically decreases plant diversity (e.g. Skarpe 1990; Angassa 2014).

### Surface fuel biomass and fire intensity

Even though fuel loads were twice as high in fenced plots, our maximum surface fuel mass recorded was low [310 g m^−2^ compared to ~ 1500–1900 g m^−2^ annual grass production in mesic *Hyparrhenia* savannas (FAO 2017), or ~ 700 g m^−2^ in mesic *Andropogon* savanna (Savadogo et al. 2007)]. One possible explanation might be that grasshoppers (*Dociostaurus* spp.), which were observed in high densities inside fences during burning experiments, consumed a substantial amount of grass biomass (c.f. Belovsky and Slade 2018).

### Tree regeneration, fire effects on trees, saplings and shrubs

Tree seedlings were generally few, but were more numerous in the fenced plots. In community exclosures in Ethiopia, regeneration success of valuable native tree species has been poor (Mekuria et al. 2018; Gebre et al. 2019; Manaye et al. 2019). Without grazing protection, palatable tree seedlings will be consumed. Chemically defenced species, such as *Combretum* spp., *Grewia* spp. and *Dombeya torrida* can become invasive in hard-grazed savannas (e.g. Gordijn et al. 2012). Acacias thorns are soft in young seedlings and these were consumed by cattle (personal observation). Restricted patches of spiny vegetation may act as grazing refugia, allowing palatable tree species to grow out of grazing height (c.f. Aerts et al. 2006). But this would require a number of years without fire. All tree seedlings died in our experimental fires.

The higher fire intensities inside our fences did not top-kill the mature trees, but did cause stem-pruning of the trees, further elevating their canopies. Only a few ~ 3–4 m tall acacia saplings in the open site with highest fire intensity were top-killed. Under dense tree canopies, there was less C4 grass, and therefore, poorer surface fuels and lower fire intensity (personal observation). In Kenya, livestock exclusion also increased fire intensity, but there, fire intensity was higher under the trees due to leaf litter accumulation (Kimuyu et al. 2014).

After our burns, shrub shoot and root mortality were higher in fenced plots, due to the higher fire intensities. According to our respondents, and personal observation, such high fire intensities are nowadays rare; because of overgrazing, there is a lack of grass fuels, and this creates “*cool fires*”. This, they explained, has made it more difficult to control *Dicrostachys* by fire, as they used to do. *Dicrostachys cinerea* is a well-known species causing shrub encroachment (e.g. Moleele et al. 2002; Hagenah et al. 2009).

### Grassland–woodland mosaic dynamics over time

Continued fire and grazing exclusion at our experimental sites could further increase fuel loads. Since fuel quantity is also controlled by herbivores, it is the combination of fire frequency and grazing pressure that control the competitive outcome between woody species and grasses (*cf.* Eckhardt et al. 2000). When grazing is suddenly removed, as happened in Gibe valley after the 1980s first Trypanosomiasis outbreak, fuels accumulate and high-intensity fires may top-kill even the large tress, as our respondents described. A similar top-kill event probably occurred in the Serengeti after the 1890s Rinderpest epizootic, triggering a regeneration pulse of acacia trees (Holdo et al. 2009). In the steep river gorge of Gibe Valley, topography is highly important for fire behaviour. Highest intensities are attained when the fire is moving uphill (Smit et al. 2013). This is probably also another mechanism that has shaped the heterogeneous savanna-woodland mosaic dominated by open grasslands, with less flammable woodlands in topographically fire-protected sites (cf. Beckett and Bond 2019).

### Landuse history and objectives

We don´t know the long-term vegetation history of the Gibe river gorge. In aerial photographs from 1957 and 1973 (Fig. S6a, b) there were larger burns and fewer large trees than today, but according to our interviews, and other studies (e.g. Reid et al. 1997), many large trees disappeared since the 1980s. Our ring counts on large acacia stumps (20–30 cm DBH) gave an estimated age of ~ 70–80 years. Based on the relatively high rainfall, Reid et al. (1997) suggested that the open grasslands might have been derived from more closed woodlands by anthropogenic burning, probably during hundreds of years. Anthropogenic burning may be even more ancient in Gibe valley. Hunter-gatherers populated many river gorges in Ethiopia in the past, and they probably used fire to increase game forage (McCann 1999). Cattle herding may also have been present since long time, due to the previous absence of Trypanosomiasis.

Landusers’ management objectives are the same today as historically; to maintain large expanses of vermin-free, grass-dominated pasture, with patches of large old trees, for shade, honey, timber and fuelwood. At the same time, the national goal is, partly contradictory, to increase tree cover and carbon storage (Lemenih and Kassa 2014). Because Ethiopia has little forest cover, increasing forests is assumed to increase biodiversity. But it takes time to recruit new old trees, and there is an inevitable young phase of spiny scrub at start. This is the least preferred vegetation type by the landusers, and increased shrub cover may also increase tse–tse prevalence (Sciarretta et al. 2005). In other shrub-encroached savannas, when grass production became insufficient for cattle grazing, pastoralists switched to shrub-browsing goats or camels, and charcoal production from large acacias, contributing to further shrub encroachment, locking vegetation into an early-successional state (c.f. Angassa and Oba 2008; Filipova and Johanisova 2017). Exclusion of fire and grazing to promote tree recruitment should be done in strategic places where new forest vegetation is needed. It cannot be done simultaneously across the whole park, due to the increased wildfire risk and conflicts with local landusers.

## Conclusions and management recommendations

In Africa, protected areas need to handle potentially conflicting goals of carbon storage, watershed management, biodiversity conservation and sustainable local livelihoods, in order to succeed with any of the goals. In contrast to other continents, where traditional fire management was terminated earlier, traditional fire mgt and grazing is still widespread in Africa. The current focus on carbon storage promotes fire and grazing exclusion over large areas. This may lead to loss of livelihoods and increased wildfire risk. This is particularly important in the face of a changing climate (Russell-Smith et al. 2015). For systems with a long history of fire and grazing, we hypothesize that after fire and grazing exclusion, there will initially be a phase of fuel build-up. Yet, if fire is excluded for a longer period, flammability might decrease with the closure of the tree canopy, depending on the flammability of the tree species and their litter. Early-season prescribed fire can increase long-term carbon storage, by reducing fuel loads, preventing late-season high-intensity wildfires and thereby ensuring survival of the large trees (Russell-Smith et al. 2015; Khatun et al. 2016). In our case, due to a long history of fire and grazing, the Gibe River gorge vegetation is adapted to this management regime. The park managers understand the importance of active fire management, but burning vegetation is nationally banned. This complicates implementation and cooperation with local communities. To protect old-tree carbon pools and biodiversity, as well as local livelihoods, we suggest a community fire management programme, making use of the still existing local traditional fire knowledge.

## Electronic supplementary material

Below is the link to the electronic supplementary material.

Supplementary material 1 (PDF 7134 kb)
