# Supplementary material for: Ecosystem restoration in fire-managed savanna woodlands: Effects on biodiversity, local livelihoods and fire intensity
Source: Ambio. 2020 May 25;50(1):190–202. doi: 10.1007/s13280-020-01343-7 (PMC7708610; doi:10.1007/s13280-020-01343-7)
Supplement: Supplementary file 1 — Supplementary material 1 (PDF 7134 kb) [file 13280_2020_1343_MOESM1_ESM.pdf]

Ambio

Electronic Supplementary Material
This supplementary material has not been peer reviewed.

Title: **Ecosystem restoration in fire-managed savanna woodlands: effects on biodiversity, local livelihoods and fire intensity**
Authors: Maria Ulrika Johansson, Firew Bekele Abebe, Sileshi Nemomissa, Tamrat Bekele, and Kristoffer Hylander

MATERIALS AND METHODS

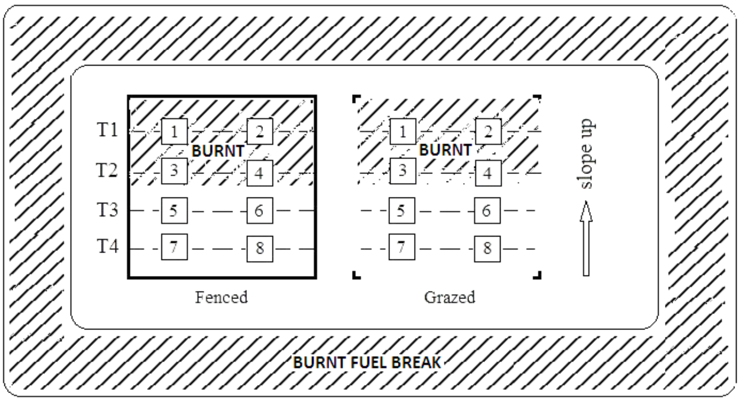

Fig. S1 Experimental design of each of the six livestock exclosures, with adjacent control plots. Each site is surrounded by a burnt fuel break. Canopy cover line transects, field layer quadrats, and upper half burn treatments

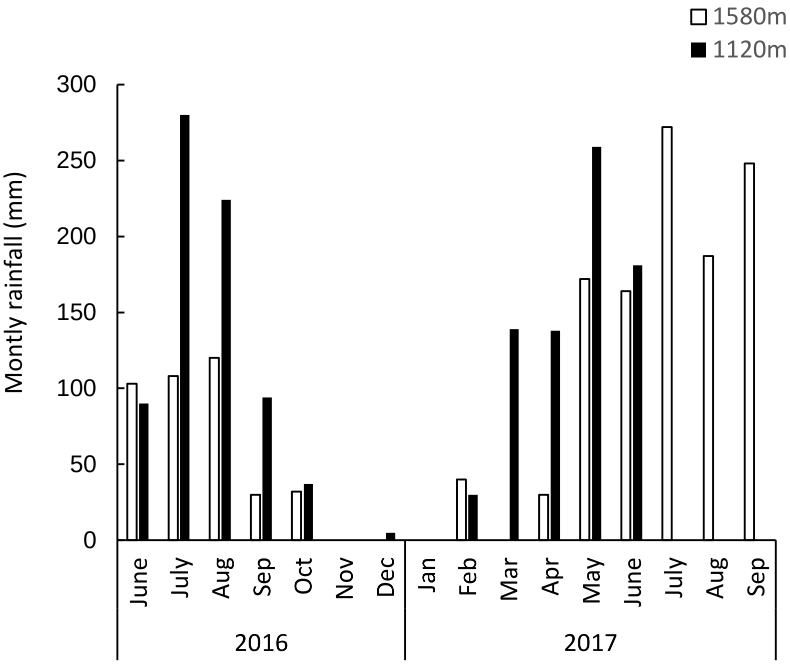

Fig. S2 Locally recorded rain data for 1120 and 1580 m.a.sl., June 2016 to Sept 2017. Annual rainfall (June–May) at 1120 m =1295 mm, and at 1600 m = 635 mm. (July-Sep for 1120 m missing data)

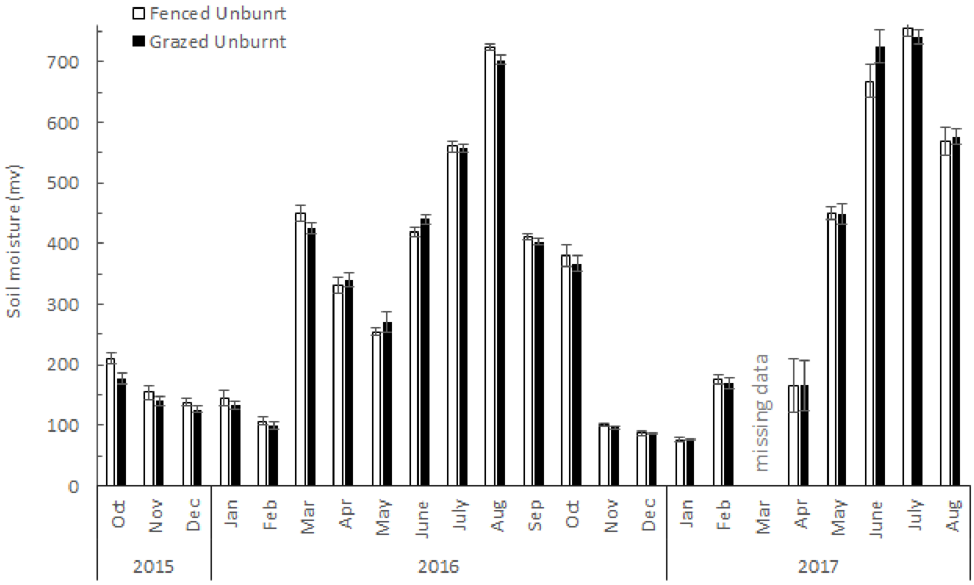

Fig. S3 Average monthly soil moisture in fenced and grazed plots. Error bars = 1 SE

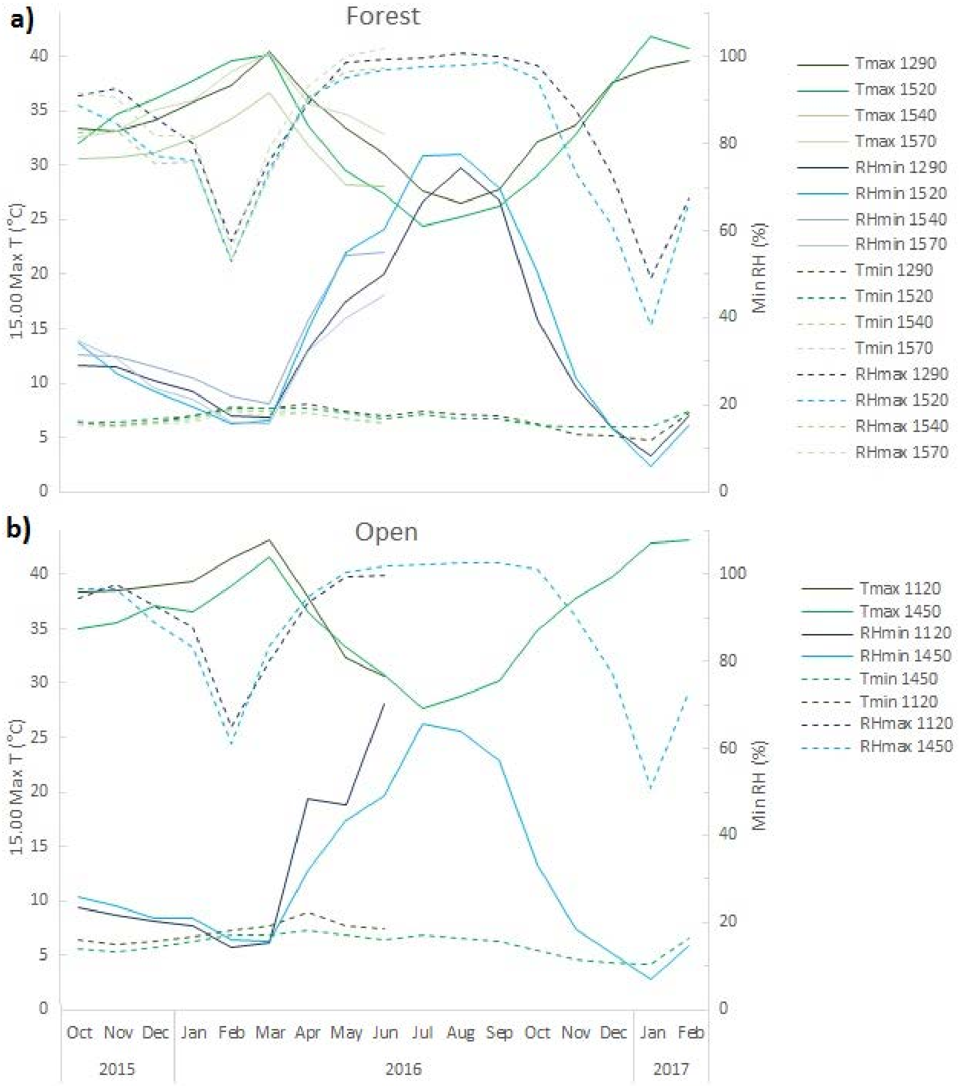

Fig. S4 a-b Monthly average $T_{max/min}$ and $RH_{min/max}$ at **a)** forest and **b)** open sites at different altitudes. Open sites had slightly higher max/min values, see **Fig. S4c**

c)

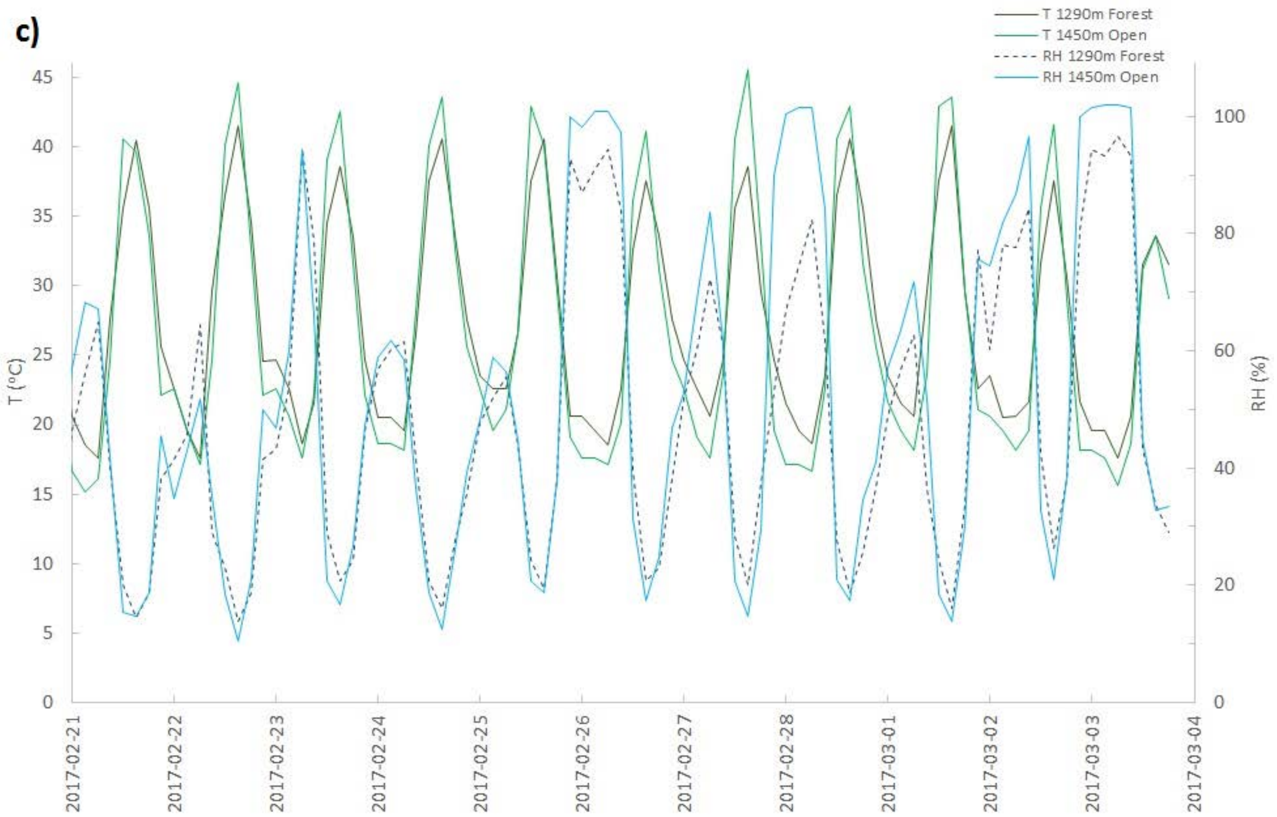

Figure S4 c) Daily Temperature and RH fluctuation during burning experiments at one forest and one open site

RESULTS

Table S1a Nested anova results for October field layer cover and height in grazed and fenced plots

		log bare soil cover		log field layer cover		grass cover		log field layer max height		log field layer av. height	
	DF	*T*	P	*T*	P	*t*	p	*t*	p	*t*	p
Treatment	1	**7.02**	**<0.001**	**-3.37**	**0.020**	**-5.13**	**0.004**	**-8.63**	**<0.001**	**-8.72**	**<0.001**
Site	5										

Significant results in bold (p<0.05). n=96, DF=degrees of freedom, some values were logged to ensure equal variance

Table S1b. Nested anova results for December surface fuel biomass in grazed and fenced plots

		log DW grass/herb litter		DW leaf litter		DW wood litter		log DW fine litter		DW total biomass	
	DF	*T*	p	*t*	P	*t*	p	*t*	p	*t*	p
Treatment	1	**-3.49**	**0.025**	0.69	0.530	-0.03	0.978	-2.17	0.096	**-2.84**	**0.047**
Site	4										

Significant results in bold (p< 0.05). n=50, DF=degrees of freedom, DW=dry weight, some values were logged to ensure equal variance

Table S2 Nested anova results for grass species richness in grazed and fenced plots

		no. of species		no. of grass species	
	DF	*T*	P	*t*	p
Treatment	1	0.00	1.000	**-4.24**	**0.008**
Site	5				

Significant results in bold (p< 0.05). n=96, DF=degrees of freedom

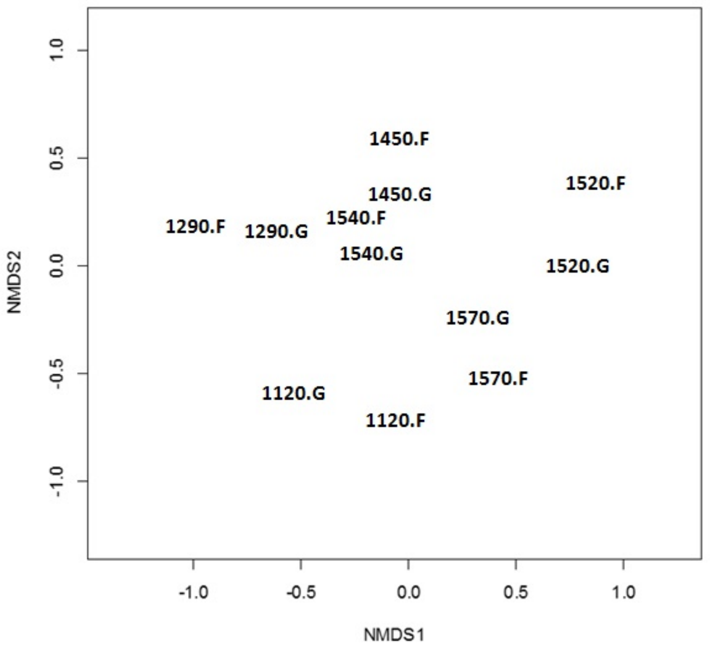

Fig. S5 Adonis results of field layer species composition in fenced (F) and grazed (G) plots at different altitudes (p=0.022)

Interview results

All respondents rely on both animal husbandry and agriculture today, with agriculture increasing in share due to land shortage and Trypanosomiasis. Elder respondents who lived their whole life in the area said that in their childhood, their parents did not practise much farming but had many more cattle, grazing freely in the river gorge. Before the first Trypanosomiasis outbreak in the beginning of the 1980´s, rich families had up to 200 cattle, for meat and milk production. Today respondents had up to 10–15 cows, 1–2 oxen, one donkey or mule, no sheep, some chicken. Only few kept goats due to risk of loss to leopards and hyenas. Livestock are herded by children in the river gorge during daytime and kept near the house at night. They continue grazing dead grass throughout the dry season until it is burnt in March–April. After crop harvest (November–December) livestock also graze farm fields. Some respondents just started supplementary feeding cattle with crop residues due to shortage of pasture. Palatable tree species are pollarded for leaf fodder. *Stereospermum kunthianum* was said to be the most important fodder tree, but not regenerating, and never observed fruiting. Vegetative propagation by one respondent failed. At lower altitudes the main crops were; sorghum, maize and chick peas, on the plateau; teff, maize, enset, coffee, fruit trees. All respondents had lost livestock to due to Trypanosomiasis lately, and said this is a serious livelihood threat. Earlier a few respondents received free medicine and help to build fly traps, but now the project is terminated and the medicine is expensive. Some of the poorest farmers had lost their last oxen and could no longer plow their fields.

All respondents experienced increased degradation of the river gorge vegetation since the 1980´s, specifically in terms of pasture productivity and shrub encroachment. Shrub encroachment, they explained, is a serious threat to their livestock by limiting access to grass and harming the cows´ udders. They were concerned by the loss of large trees, which they said started with the first bridge construction in the 1940´s, and increased with the 1980´s resettlement programmes, charcoal production, agricultural investors, and population growth. Many respondents said that the loss of large trees has made dry seasons hotter and dryer, and reduced honey production and construction timber supply. All respondents supported park objectives to protect the forest and biodiversity and some were expecting

income from tourists in the future. But many were also increasingly negatively affected by wildlife from the park, e.g. hippos and baboons raiding crops, and hyenas and leopards taking goats and calves.

No respondent had received compensation for loss of access to pastureland. Since they need pasture, they were not able to abide to the general grazing ban, and it was unclear whether the grazing ban was total, or if there was a penalty fee for breaking the rules. They supported the new bans on tree cutting and bark harvest. Both still occur, but at lower rates than before park establishment. Many respondents said they support the general burn-ban because fire in nature is only bad. At the start of interviews, many said that fires are accidentally lit during honey collection, or by fishermen to clear paths. But later they said that without fire there would be less grassland and more shrubs, and that this would be bad for their livestock. They said that some people deliberately start the fires, mainly to reduce wildlife attacks on livestock and people, by the giant python, hyenas, leopards (and in the past also lions), but also to remove encroaching shrubs with habitat for the tse-tse flies.

The first outbreak of Trypanosomosis ("*Gendi*"), was the major historic catastrophe. It killed almost all livestock, as one respondent said: "– *Not a cow, not a goat, not even a chicken was to be found in the whole area …*" Another respondent said, "*after Gendi there was a big forest fire, and 56 houses were burned, but no man died*"… *"many big trees died*". After Trypanosomiasis arrived, respondents on the North-Eastern plateau reduced their dependency on cattle and intensified enset/coffee agroforestry and planted exotic fruit trees supplied by extensionists. Respondents on the drier and lower South-Western side of the river reduced herd sizes and increased cultivation of maize and sorghum, now mainly using oxen as draught power. So do the more recently resettled farmers do at the lowest altitudes near the river.

Most respondents could easily identify from photos the types of vegetation which produced the best pasture. The cattle prefer the short grass species, the long grass is only used for thatching. The more grazing, the more the short grass species dominate, as well as suffrutescent unpalatable herbs, according to some respondents. The goats browse some of the herbs the cattle reject. All respondents said that shrub encroachment has increased, and that the shrubs can be killed by fire. Most said that increased grazing pressure has reduced the amount of remaining grass in March–April, and that this has caused the "*cool fires*" to be more common, and these do not properly kill the *Dicrostachys* shrubs and young acacias. Especially the roots are difficult to kill, but it is easier at the end of the dry season, when shrubs are very dry, according to one respondent. It is also best to burn just before the rains in April, so the ash will rejuvenate the new grass, according to some respondents. Many respondents said that the agricultural bureau had taught them that fire is bad because it causes deforestation, and some said that the grass can rejuvenate as well without burning. They said that fires used to be more high-intensity in the past, and then killed more shrubs. Elder respondents could correctly identify most tree species in the photos, and best explained the importance of wind, terrain and surface fuels, e.g. that fires stop at forest margins mainly due to the lack of grass fuels. Deep fire management knowledge was found mainly among respondents with pastoralist background, e.g. in how they could explain how they controlled fire behaviour in tall grass in the past: by burning down-hill, or on flat land in the evening, with 5 men igniting in a ring formation, easily controlling the backing short flames at the outer perimeter. Resettled agrarian respondents said that in the north where they came from there was no landscape burning because there was no wild forest. Some cited an agrarian proverb about how fire is never good on the landscape, only in the stove: *"There is no good fire, from good fire the thief is nicer"* (meaning that the thief only takes some of your things, the fire takes it all). They used fire only to clear weeds in the croplands and to try to eradicate *Dicrostachys*.

Table S3 Plot data and Tree and Shrub cover (%) (average in 30 m line transects, n=4)

Site altitude (m.a.s.l.)	1120		1290		1450		1570		1520		1540	
Coordinates (UTM)	N8°13.936' E37°35.070'		N8°15.352' E37°34.866'		N8°15.833' E37°36.622'		N8°14.667' E37°37.585'		N8°15.076' E37°37.208'		N8°14.347' E37°38.110'	
Slope (%) and aspect	4% S		1% N		0.2% S		4% W		2% S		3% SE	
Catena position	deposition		deposition		deposition		erosion stony		erosion stony		erosion stony	
Soil Type	vertisol		vertisol		vertisol		nitisol		nitisol		nitisol	
Canopy height (m)	6	5	10	10	4	5	5	5	6	5	11	8
Basal area (m^2 ha^{-1})	3.8	2.3	10.2	6.0	1.9	2.0	7.0	6.0	5.7	7.5	7.9	8.1
Treatment	F	G	F	G	F	G	F	G	F	G	F	G
**Tree species**												
Acacia etebaica			22	32	8	17					22	
A. nilotica				27						1	24	27
A. polyacantha	20	17				5						
A. seyal			29		7	5	3				23	20
Combretum collinum subsp. binderianum	12						7	23	19	21	5	14
C. molle			23		3			4	10	11	16	10
Cussonia holstii	20	2							17	7		1
Rubiaceae sp.		17										
Dalbergia lactea				4								
Dombeya torrida								6	12			
"Enefo" (Gur.)							13					
Grewia mollis	5			7	4	5				13		3
"Sudan" (Gur. Am.)			3	13								
Maytenus senegalensis		5					9	10	7	7		
**Tree cover %**	**57**	**41**	**77**	**83**	**22**	**32**	**32**	**43**	**65**	**60**	**90**	**75**
Av. # tree ind./trans.	1	1	2	2	3	4	3	3	4	5	4	3
Av. # tree sp./trans.	1	1	2	2	2	2	3	2	3	4	2	2
**Shrub species**												
Dicrostachys cinerea	11	12	9	9	18	12	12	12	10	14	12	12
Fluegga virosa	16	19	3	5								
Searsia natalensis*					5	10	5	10		3		12
Ehretia cymosa	2											
Ximennia americana					10							
Rubus apetalus									24	11		
Ziziphus abyssinica					3							
**Shrub cover %***	**29**	**31**	**12**	**14**	**36**	**22**	**17**	**22**	**34**	**28**	**12**	**24**
Av. # shrub ind./trans.	5	4	2	2	5	5	3	2	2	4	1	2
Av. # shrub sp./trans.	2	2	1	1	2	2	2	2	1	2	1	2

F=Fenced, G= Grazed, *If tree species were <250 cm (uncommon) they were recorded in the shrub layer. Trees in "*italics*"=local names. **Searsia natalensis* (former *Rhus natalensis*)

Table S4 Field layer cover (%) per site and treatment, Fenced (F) & Grazed (G), frequency and indicator species

	1120 m		1290 m		1450 m		1570 m		1520 m		1540 m		#		
	F	G	F	G	F	G	F	G	F	G	F	G	plots	Ind.	p
Abutilon figarianum	5	5			5	5	5	5	10	7	10	5	20		
Acacia abyssinica									10	5			2		
A. etebaica					5								3		
A. nilotica									5				1		
A. polyacantha			10	5	7	5							7		
A. seyal										5			1		
Achyranthes aspera	17	18	8	5	8	10	10	5	5	12	10	7	29		
Ageratum conyzoides	10	20	35	10	13	38	7	16	14	14	13	19	70	G	0.07
Bidens pilosa	20	5	10	5	10	15	15	15	18	10	23	12	43		
**Bothriochloa insculpta**	**10**	**5**	**10**	**5**	**15**	**5**	**10**	**5**	**5**	**5**	**10**	**5**	**12**	**F**	**0.02**
Celosia argentea									10	5			2		
Crotalaria plowdenii									10	10			2		
Cynodon dactylon					13	10							3		
Dicrostachys cinerea	5												1		
Dombeya torrida							5						1		
Dregea schimperi	5						5	5					3		
Glycine wightii			35	5					5	10			5		
Grewia mollis			5										1		
Heteropogon contortus	25	15	15	25	25	11	21	10	17	9	16	10	43	F	0.06
Hvpoestes triflora								10	9	11	5	15	13		
Hygrophila schulli					5	5	5	5	5	5	5	5	15		
Hyparrhenia cymbaria					16	15	11	10	25	10			22		
H. dregeana					28	5	13	5	10	5	25	10	14		
H. filipendula	30						16	5					8		
Hypoestes forskalii			36	19	17	11	8	9	10	10	19	19	65		
Justicia heterocarpa	8	5	19	7	6	6	8	8	5	5	11	6	53	F	0.24
**Leucas deflexa**	**7**	**5**	**8**	**16**	**8**	**9**	**5**	**11**	**5**	**10**	**9**	**10**	**45**	**G**	**0.04**
Melinis repens									20	5	5	5	4		
Ocimum urticifolium	10						10	5	5	10			5		
Oplismenus hirtellus					20	5							2		
Panicum maximum											10		1		
Parthenium hysterophorus	5						5	15					4		
Pennisetum purpureum	47	5	20	5	16	5							14		
P. trachyphyllum					20	5							2		
Peristrophe paniculata	13	5	10	15	10	7					8	10	14		
Senna obtusifolia	15	14									10	10	8		
Sida rhombifolia					5	5			13	20			6		
Sorghum arundinaceum	55		10	5	10	5	15	5	10	5			10	F	0.12
S. purpureo-sericeum	20	10											2		
Tagetes minuta					10	5							2		
Triumfetta rhomboidea	15						29	13	10	5	10	5	20		
Vernonia congolensis	15						15	10					3		

plots = number of subplots in which the species was recorded (n=98), Ind. = Indicator species for treatment, F = Fenced, G = Grazed, p = p-value for indicator species

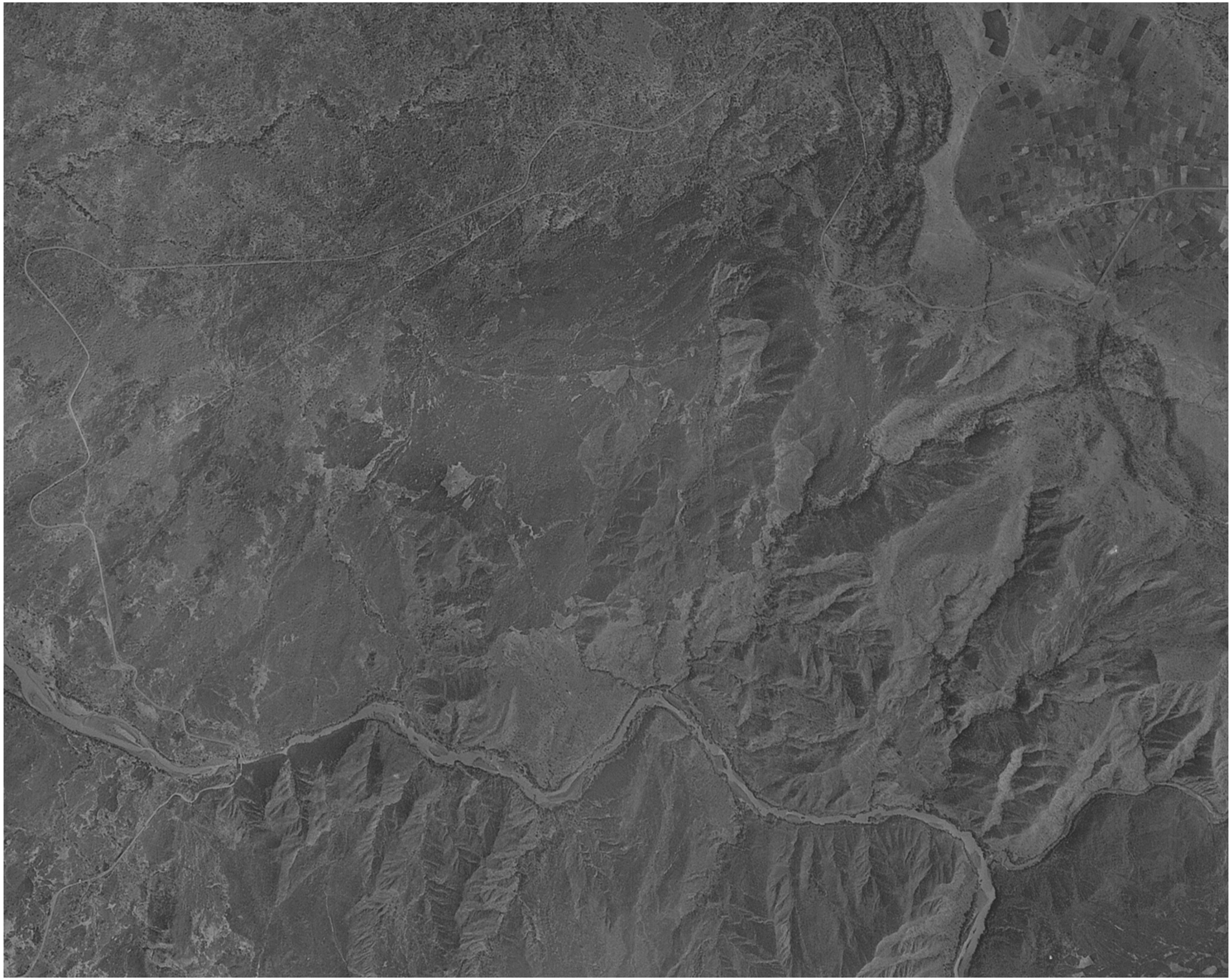
Fig. S6 a) Study Area between Jimma road and Gibe river in December 1957, large dark-colored area in center is a fresh burn

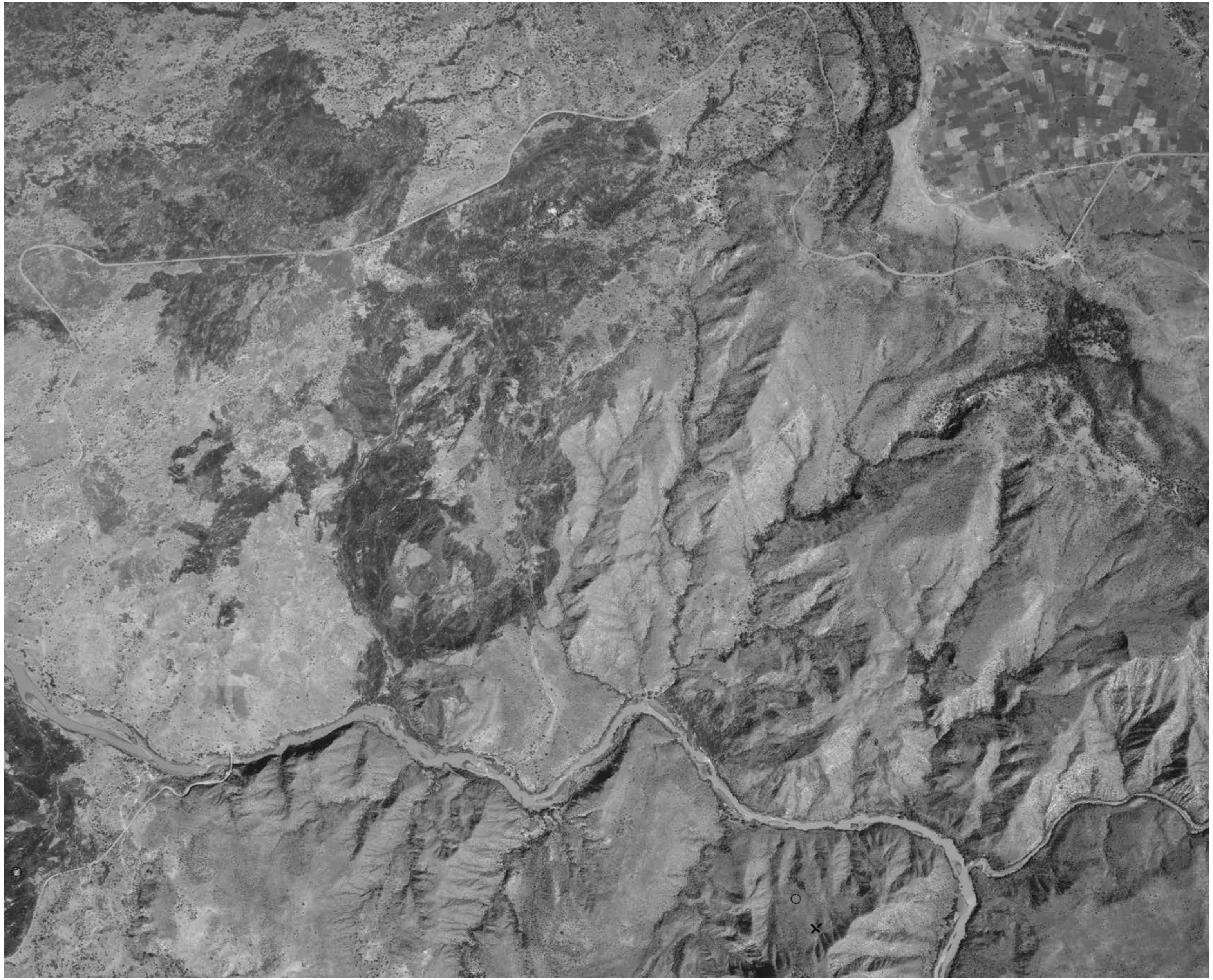

Fig. S6 b) Study Area between Jimma road and Gibe river in December 1971. large dark-colored areas in center are fresh burns

APPENDIX 1 INTERVIEW QUESTIONS & PHOTOS

General Questions:

A. Name? B. Age? Gender? C. Education D. Since when did you live here? Why moved? E. #Children? F. #Cows? #Oxen? #Goats? #Donkeys? #Sheep? #Chicken? F. Which crops do you grow? G. What % of your income comes from Cows? Goats? etc., Agriculture?, Off-farm activities?
H. What are the largest differences between today, and when you were a child/came here?
I. How did this place look like when you were a child/first came here, compared to now?
J. Is there a difference in landuse/land cover - past to present?
K. What are the uses of the Gibe river gorge?
L. What has happened to family sizes over time?
M. What is the relation between people and forest – over time?
N. Proportional area cover of grass – trees / grass – herbs over time? How to improve pasture?
O. What are the reasons for burning?
P. Fire management techniques? How? Has this changed over time?

Photo questions:

**Question:**	**Follow up question (depending on answer):**
1. What is this?	(*Acacia*, flat land, fence, person) Good pasture? Why? How old trees?
2. What is this?	(*Combretum* slope) is this good pasture? Why?
3. What is this?	(*Combretum* flat land, herbs) possible to improve pasture? how? Why more herbs?
4. Is this good pasture?	(flat land, *Cussonia*) is it possible to burn? why? which conditions are required?
5. Is this good pasture?	(burnt ground, resprouting grass) Good pasture? Why? Effects of fire? Herb/grass?
6. Is this good pasture?	(bush-encroached, long grass) how old? can it burn now?
7. Is this good pasture?	(flat land, long grass) Why? Which grass species best? Large/small Hyparrhenia?
8. What is this?	(*Stereospermum*) Cows eat leaves? Which trees cows prefer? Why no seedlings?
9. What is this?	(large *Acacia polyacantha*) How old is it? Why are large trees rare today?
10. What is this?	(inside green fire refugium) Why greener around streams?
11. What is this?	(Fire refugium seen from above) Why is this not burnt?
12. What is this?	(riverine forest) Why green along river?
13. What is this?	(fire refugium in crater) Why is this not burnt?
14. Is this a good burn?	(long grass fire) Why did some patches not burn? ignition tools? Technique? Wind?
15. Is this a good burn	(Dec long grass fire, man) Under what conditions is it easy to burn tall grass?
16. Is this a good burn	(Feb long grass fire, men) Which time of year burn? Why? When is wind direction best?
17, What is this?	(burning leaf litter) Which is better fuel dry grass or dry leaves?
18. Is this a good burn?	(Landscape fire) where is wind? where ignited? which wind preferred? What happens if wind change? accidents? someone killed? how do you prevent danger?
19. Is this a good burn?	(Oromia fire) why? is there a difference in total area burnt each year, why? possible to burn during night? does fire move faster uphill/downhill? who is burning, men, women, children? what is the legal situation? can you get permission to burn? how? can lightning start fire?
20. Is this a good burn?	(close-up fire) why? How long flames kill big trees/bush? Which trees fire sensitive?
21. What is this?	(scar on base of *Acacia etebaica*) why? Can fire make this kind of scar?
22. What is this?	(*Acacia seyal* bark) Why does the bark peel off? Fire adaptation?
23. What is this?	(fence) what will happen when excluding cows? Higher fire intensity? Kill trees?
24. What is this?	(fence) what will happen with tree cover when excluding livestock? have you seen fire in closed-canopy forest? Under which conditions can forest burn?

Did you hear about the cattle disease? (*Gandi*) when did it come to Gibe? What happened to the vegetation then? Was there intensive fires after the disease?

Q. Relations between authorities and people? Relations between national park staff and people? Change over time? Did we forget to ask any question which is important for your livelihood here?

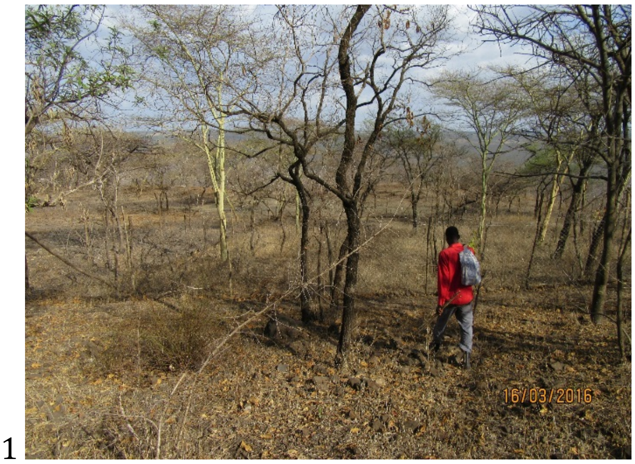

1

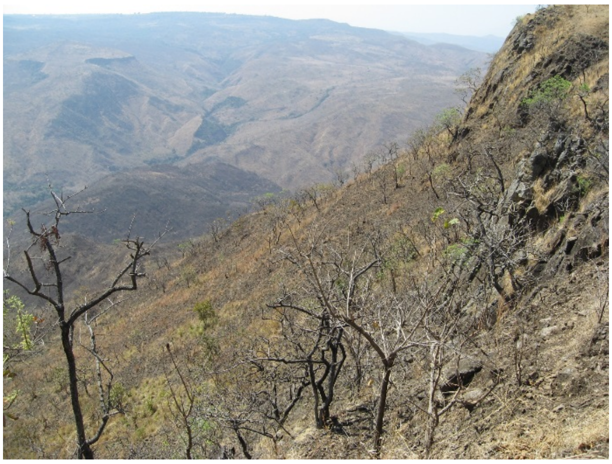

2

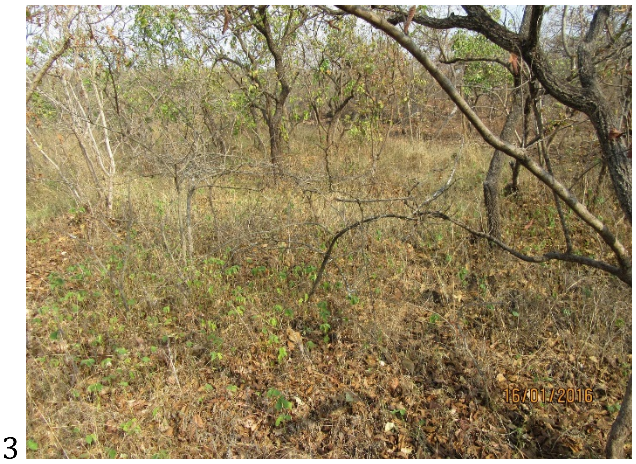

3

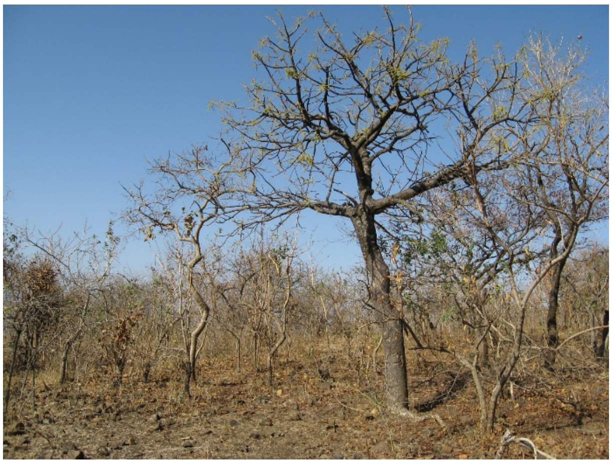

4

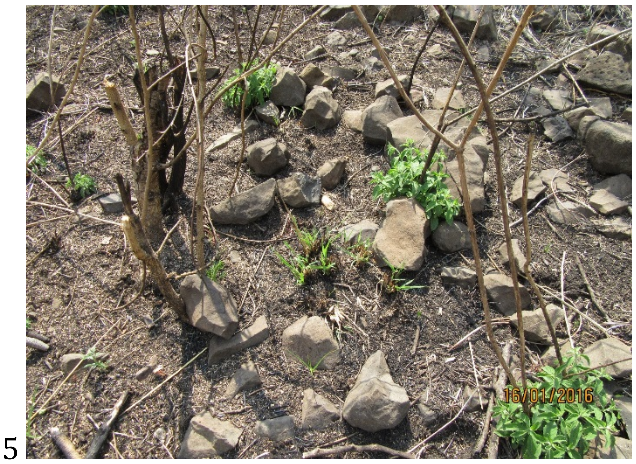

5

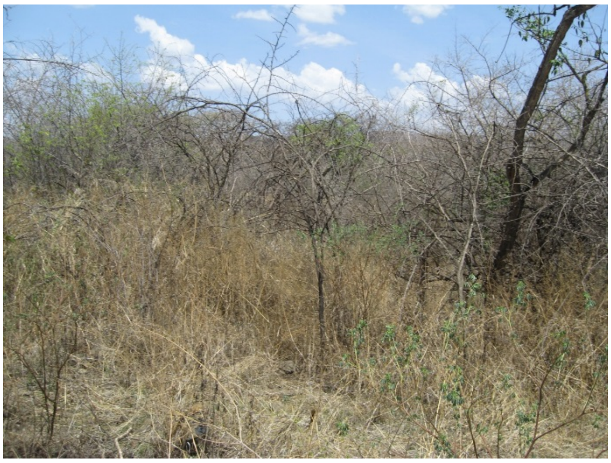

6

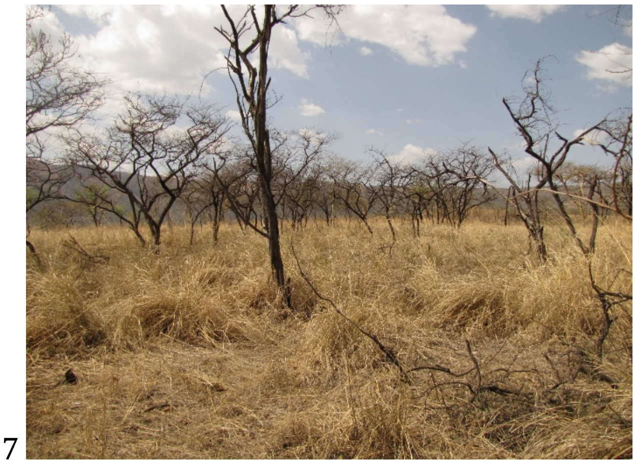

7

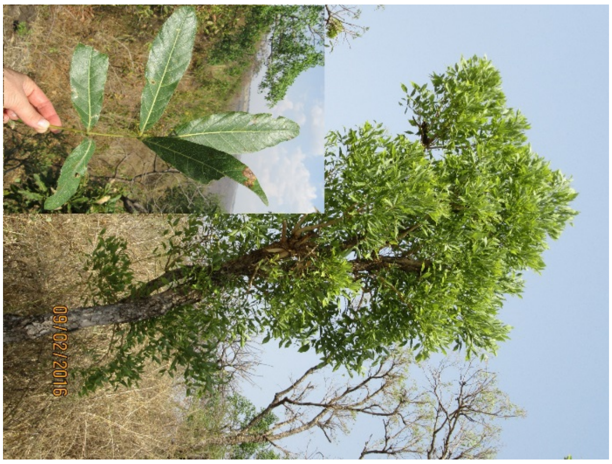

8

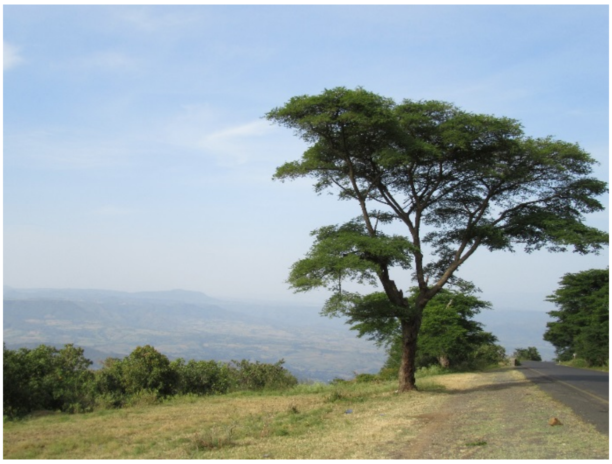

9

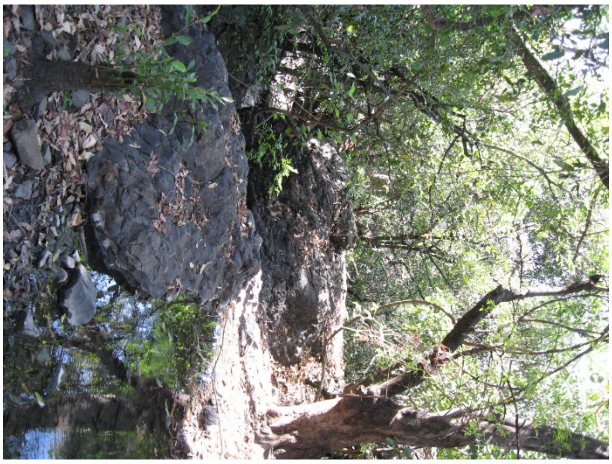

10

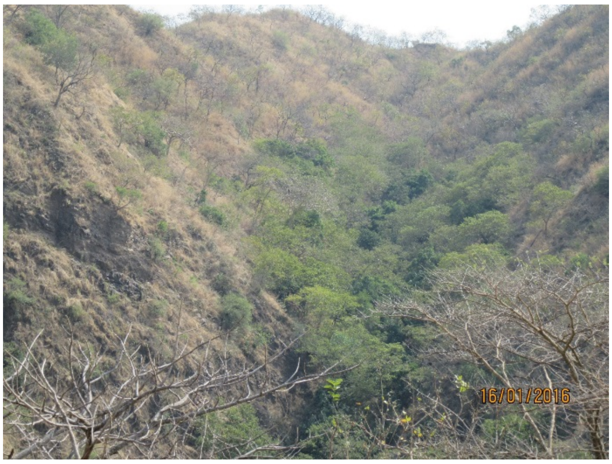

11

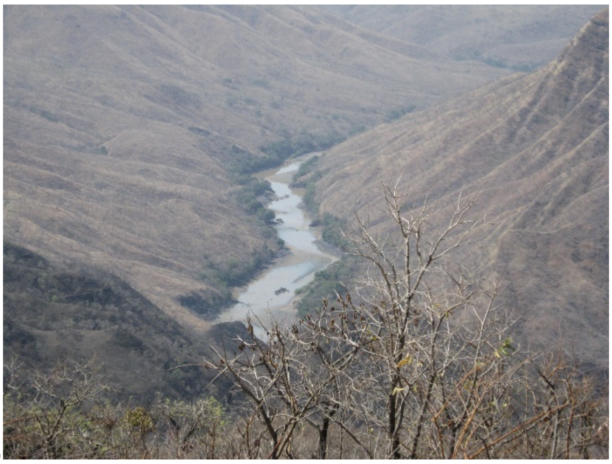

12

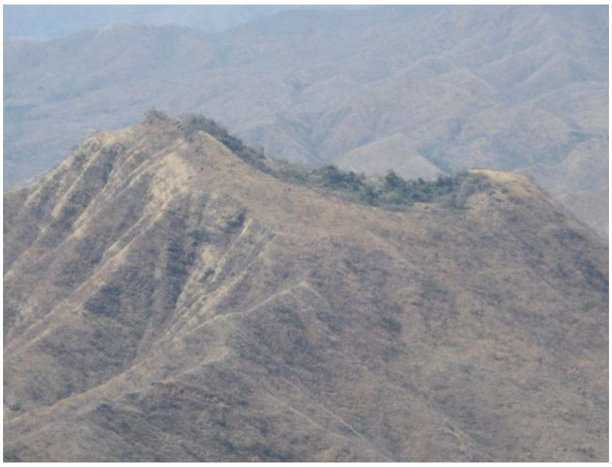

13

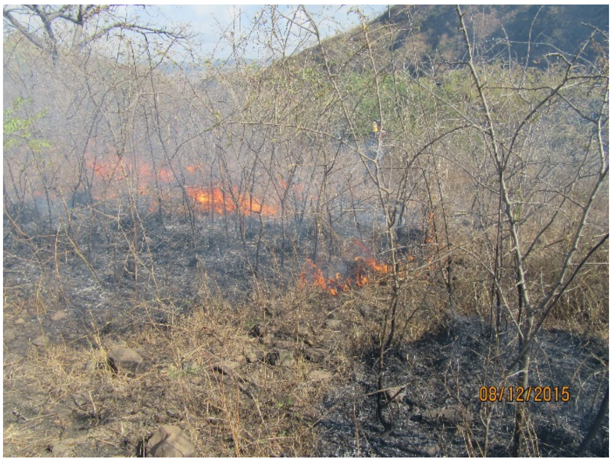

14

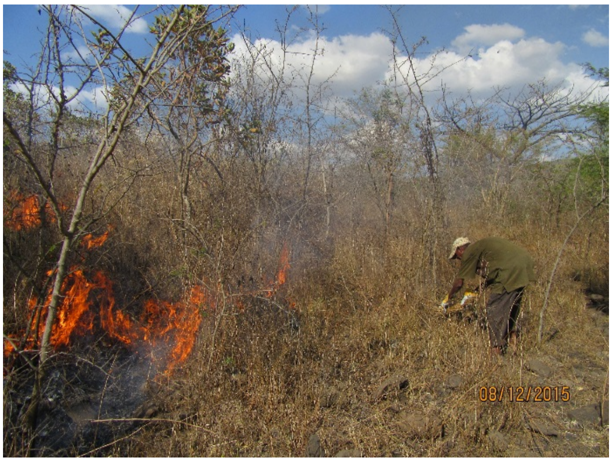

15

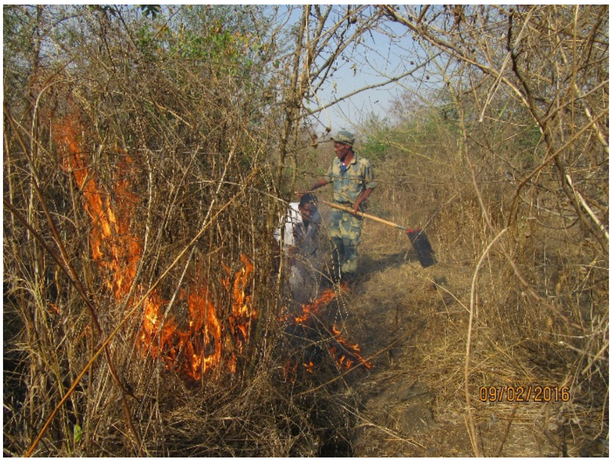

16

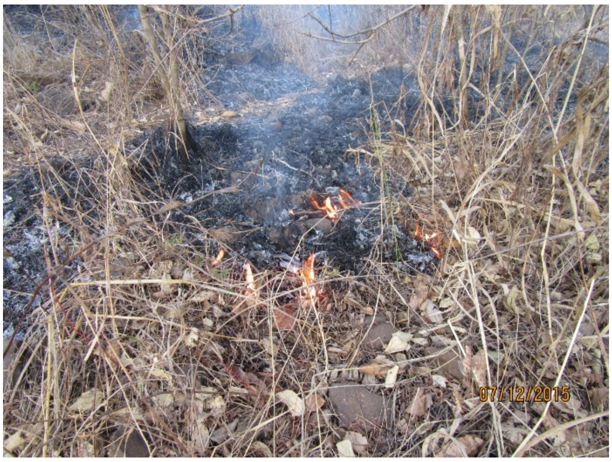

17

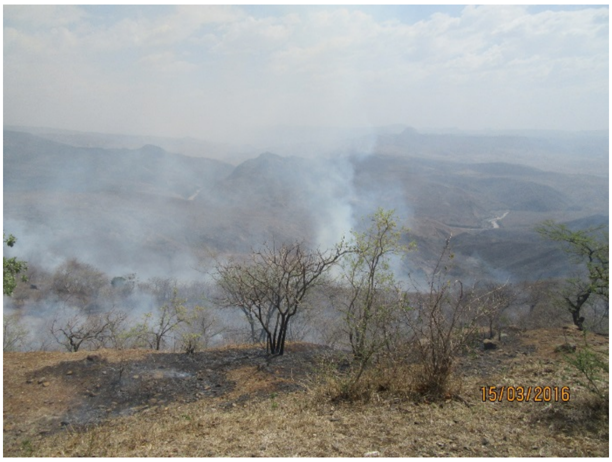

18

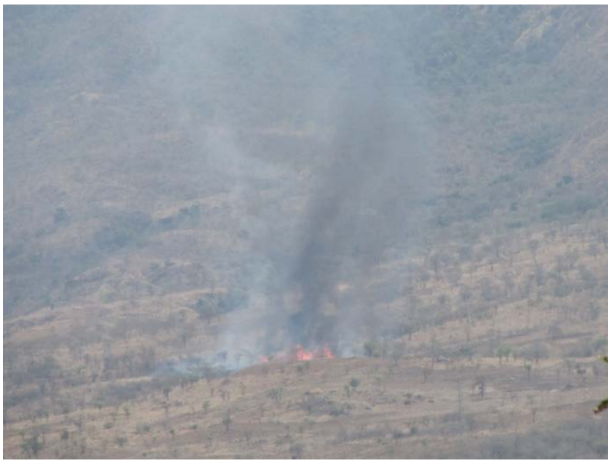

19

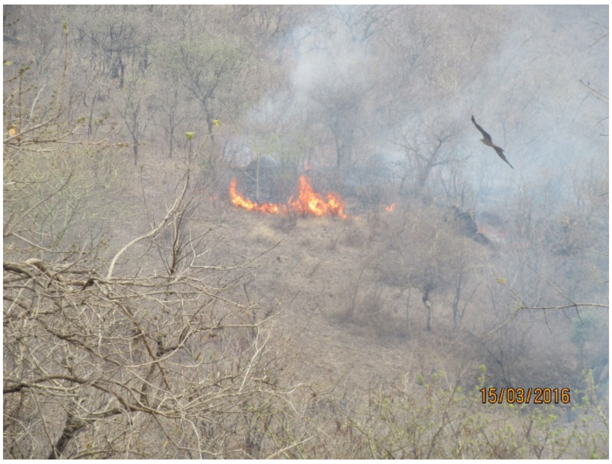

20

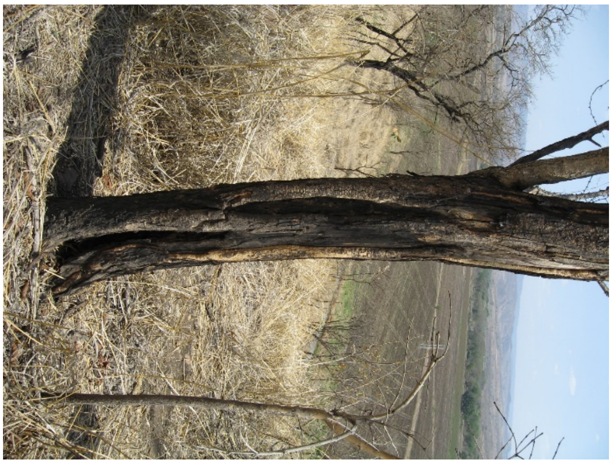

21

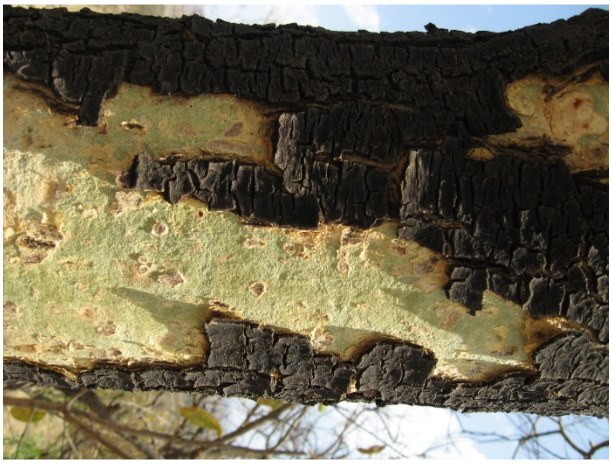

22

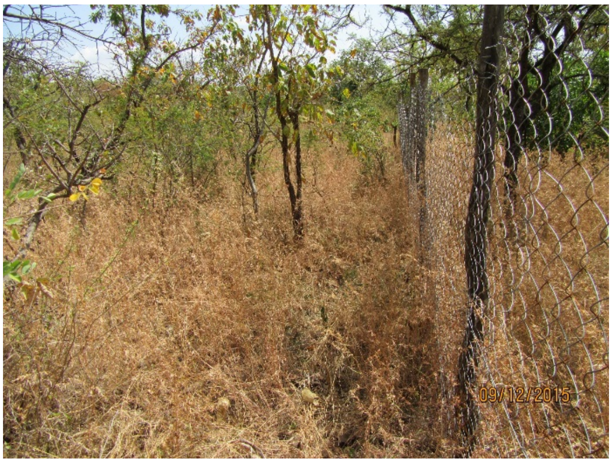

23

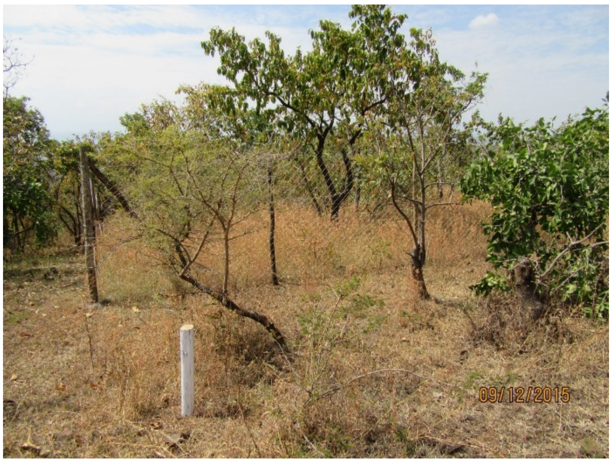

24
